# CXCL4 Links Inflammation and Fibrosis by Reprogramming Monocyte-Derived Dendritic Cells *in vitro*

**DOI:** 10.3389/fimmu.2020.02149

**Published:** 2020-09-17

**Authors:** Sandra C. Silva-Cardoso, Weiyang Tao, Chiara Angiolilli, Ana P. Lopes, Cornelis P. J. Bekker, Abhinandan Devaprasad, Barbara Giovannone, Jaap van Laar, Marta Cossu, Wioleta Marut, Erik Hack, Rob J. de Boer, Marianne Boes, Timothy R. D. J. Radstake, Aridaman Pandit

**Affiliations:** ^1^Center for Translational Immunology, Department of Immunology, University Medical Center Utrecht, Utrecht University, Utrecht, Netherlands; ^2^Department of Rheumatology and Clinical Immunology, University Medical Center Utrecht, Utrecht University, Utrecht, Netherlands; ^3^Department of Dermatology and Allergology, University Medical Center Utrecht, Utrecht University, Utrecht, Netherlands; ^4^Theoretical Biology, Utrecht University, Utrecht, Netherlands; ^5^Department of Pediatrics, University Medical Center Utrecht, Utrecht University, Utrecht, Netherlands

**Keywords:** CXCL4, dendritic cells, fibrosis, gene regulatory networks, inflammation

## Abstract

Fibrosis is a condition shared by numerous inflammatory diseases. Our incomplete understanding of the molecular mechanisms underlying fibrosis has severely hampered effective drug development. CXCL4 is associated with the onset and extent of fibrosis development in multiple inflammatory and fibrotic diseases. Here, we used monocyte-derived cells as a model system to study the effects of CXCL4 exposure on dendritic cell development by integrating 65 longitudinal and paired whole genome transcriptional and methylation profiles. Using data-driven gene regulatory network analyses, we demonstrate that CXCL4 dramatically alters the trajectory of monocyte differentiation, inducing a novel pro-inflammatory and pro-fibrotic phenotype mediated via key transcriptional regulators including CIITA. Importantly, these pro-inflammatory cells directly trigger a fibrotic cascade by producing extracellular matrix molecules and inducing myofibroblast differentiation. Inhibition of CIITA mimicked CXCL4 in inducing a pro-inflammatory and pro-fibrotic phenotype, validating the relevance of the gene regulatory network. Our study unveils that CXCL4 acts as a key secreted factor driving innate immune training and forming the long-sought link between inflammation and fibrosis.

## Introduction

Fibrosis is uncontrolled accumulation of extracellular matrix (ECM) in multiple organs and accounts for one third of deaths worldwide (1, 2). Fibrosis is considered to be a result of complex cellular and molecular interplay following tissue inflammation and injury. Across a wide range of diseases, fibroblasts inappropriately synthesize and release increased amounts of ECM components, suggesting a conceptual framework in which myofibroblast transition is the key event leading to fibrosis (1). Recent studies however, strongly implicate the innate immune system as a critical contributor to fibrosis development (3, 4), in line with clinical observations that an inflammatory phase precedes fibrosis by years. Hence, identification of the molecular pathways linking inflammation to fibrosis will provide unprecedented opportunities for drug development to treat or even reverse tissue fibrogenesis (2, 4).

CXCL4, a chemokine initially identified as a product of activated platelets, is now known to be secreted by a variety of immune cells (5–7). CXCL4 drives a broad spectrum of immune-modulatory effects in both hematopoietic stem and progenitor cells, as well as differentiated immune cells. For instance, megakaryocyte-derived CXCL4 is involved in several hematopoietic processes, including inhibition of megakaryopoiesis and maintenance of hematopoietic stem cell quiescence (8). In human CD34^+^ hematopoietic cells, knockdown of CXCL4 significantly decreased cell viability and colony forming cell potential (9). In addition, the exposure of monocyte-derived dendritic cells (moDCs) to CXCL4 during differentiation alters the phenotype and function of the cells (10–12). Moreover, studies on T-cells indicated that CXCL4 inhibits proliferation and IL-2 production on activated T-cells, induces regulatory (CD4^+^CD25^+^) T-cell proliferation while inhibiting non-regulatory (CD4^+^CD25^−^) T-cell proliferation and drives T-cell polarization (13). CXCL4 has also been implicated in the pathology of a variety of inflammatory diseases including myelodysplastic syndromes, malaria, HIV-1, atherosclerosis, inflammatory bowel disease, and rheumatoid arthritis (14–23). For example, levels of CXCL4 were significantly higher in inflammatory bowel disease and giant cell arteritis than in the non-inflammatory controls (24). In patients with early rheumatoid arthritis, mRNA and protein expression of CXCL4 were significantly elevated compared with uninflamed controls (23). Furthermore, depletion of the Pf4 gene coding for CXCL4 in Apoe^−/−^ deficient mice reduced atherosclerotic lesion formation (25). CXCL4 has been shown as a molecular mediator of liver fibrosis (26). Previously, we identified CXCL4 in pDCs as an early biomarker for Systemic sclerosis (SSc), an archetypical fibrotic disease in which endothelial cell damage and immune activation typically culminates in inflammation and fibrosis of the skin and internal organs (7).

Dendritic cells (DCs) are professional antigen presenting cells (APCs) that play crucial role of bridging innate and adaptive immune responses, by sensing danger-associated molecular patterns (DAMPs) from damaged tissues or pathogen-associated molecular patterns (PAMPs) from microorganisms by pattern-recognition receptors (PRR). Amongst them are the Toll-like receptors (TLRs), NOD-like receptors (NLRs), RIG-I like receptors (RLRs) and C-type lectin receptors (CLRs), which signaling in DCs instructs adaptive immune responses (27). Disturbed DC frequencies in circulation and in inflammatory tissues, impaired immune function and aberrant TLR-mediated responses have been associated with multiple autoimmune conditions, including systemic sclerosis (28–30).

Monocyte-derived dendritic cells (moDCs) can be differentiated *in vitro* by culturing monocytes isolated from human donors and are considered as DC model that mimics *in vivo* DC biology. Previously, we investigated whether circulating CXCL4 potentiates aberrant TLR-mediated responses and T-cell dysregulated responses observed in autoimmune diseases (14, 20). Considering the presence of CXCL4 during early inflammation and its role in modulating key immune functions, we postulated that CXCL4 might constitute the link between inflammation and fibrosis. Here we tested this hypothesis, and provide a mechanistic insight toward the molecular pathways involved in the reprograming of DC function by CXCL4. To this end, we applied a systems biology approach examining the transcriptional and epigenetic effects of CXCL4 on monocytes during and after differentiation, integrating 65 paired time courses of whole genome transcriptional and methylation profiles, and reconstructed CXCL4-dependent gene regulatory networks.

## Materials and Methods

### Differentiation and Stimulation of CXCL4-moDCs

**Figure 1:**
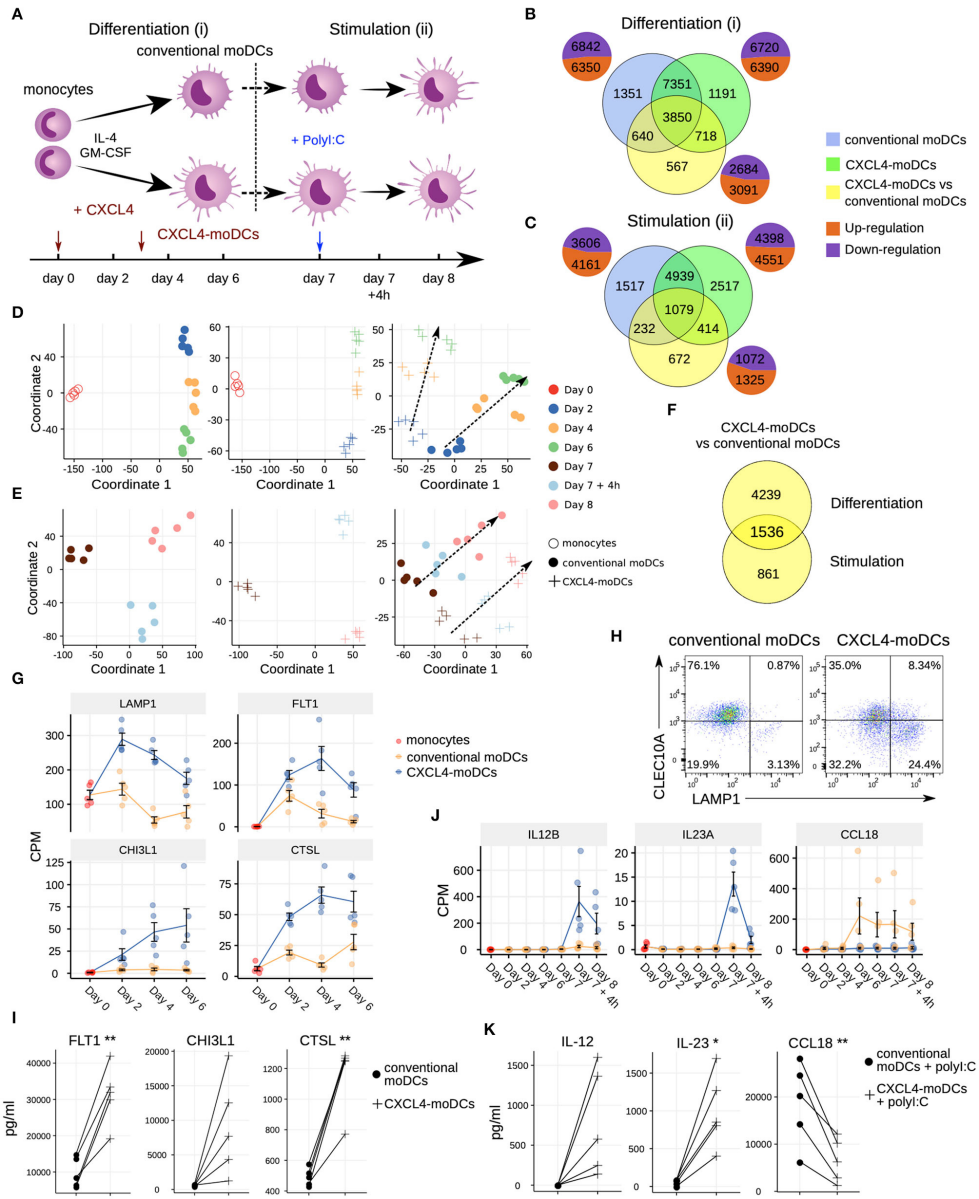
Transcriptomic programing of CXCL4-moDCs. **(A)** Schematic overview of the experimental setup: (i) differentiation of monocytes to conventional moDCs or CXCL4-moDCs; (ii) stimulation with polyI:C on day 7, for 4 h or 24 h. Overlap of differentially expressed genes (DEGs) during **(B)** differentiation and **(C)** after polyI:C stimulation of: monocytes into conventional moDCs (blue); monocytes into CXCL4-moDCs (green); and between CXCL4-moDCs and moDCs during differentiation (yellow). In **(B,C)** pie charts showing the number of upregulated (orange) and down-regulated (purple) genes. Multi-dimensional scaling (MDS) plot **(D)** differentiating and **(E)** stimulated conventional moDCs (left panel), CXCL4-moDCs (middle panel), and CXCL4-moDCs vs. conventional moDCs (right panel). In **(D,E)** dotted lines indicate trajectories over time. **(F)** Overlap of DEGs between CXCL4-moDCs and conventional moDCs, during differentiation and upon stimulation. Gene expression of example genes differential during **(G)** differentiation and **(J)** stimulation between CXCL4-moDCs and conventional moDCs. Validation of **(H)** protein expression (flow cytometry) and **(I)** cytokine production (Luminex) on day 6. **(K)** Validation of cytokine production (Luminex) on day 8. Gene expressions are shown as mean ± SEM. CPM, count per million. In panels **(I,K)**, lines connect individual donors (*n* = 5). *P < 0.05; **P < 0.01, paired two-sided Student's *t*-test.

Blood from healthy donors (HDs) was collected in accordance with institutional ethical approval. Peripheral blood mononuclear cell (PBMC) and monocyte isolation, as well as differentiation of monocyte-derived dendritic cells (moDCs) were performed as described previously (20). Briefly, PBMCs were isolated from heparinized venous blood using Ficoll Paque^TM^ Plus (GE Healthcare) density gradient. Monocytes were purified with anti-CD14 magnetic beads-based positive isolation using autoMACS Pro Separator-assisted cell sorting (Miltenyi Biotec), according to the manufacturer's protocol. Monocyte purity was above 95% for all of the samples. For the differentiation of moDCs, monocytes were cultured at a density of 1 × 10^6^ cells/ml in culture medium comprised of RPMI 1640 with GlutaMAX (Life Technologies) supplemented with 10% (v/v) heat-inactivated fetal bovine serum (FBS; Biowest) and 1% (v/v) antibiotics (penicillin and streptomycin; Life Technologies). In order to generate moDCs, GM-CSF (800 U/ml; R&D) and IL-4 (500 U/ml; R&D) were added to the culture. For the experiments where we investigated the effects of CXCL4, we added 10 μg/ml of recombinant human CXCL4 (PeproTech) on day 0 and day 3. Medium and cytokines were refreshed on day 3. Differentiated moDCs were obtained after 6 days from monocytes cultured at 37°C in the presence of 5% CO_2._ After differentiation, cells were washed, plated at a density of 0.5 × 10^6^ cells/ml and left overnight (O/N) in new culture medium. Cells were stimulated with 25 μg/ml of polyinosinic-polycytidylic acid (polyI:C; InvivoGen) for 4 or 24 h, or kept unstimulated, as shown in Figure 1A.

### 5-aza-2'-Deoxycytidine Treatment

Monocytes were seeded at a density of 1 × 10^6^ cells/ml and cultured in medium supplemented with IL-4, GM-CSF with (for CXCL4-moDCs) or without CXCL4 (for conventional moDCs), as described above. Cells differentiating into CXCL4-moDCs were either left untreated or were treated with the DNA methyltransferase inhibitor 5-Aza-2′-deoxycytidine (Sigma Aldrich) at the concentration of 100 nM. On day 3, cells were harvested and processed for RNA analysis.

### DNA and RNA Extraction for DNA Methylation and RNA Sequencing Analysis

For DNA methylation and RNA sequencing analysis cells were collected from 5 HDs: on the first day of culture (monocytes, day 0); during differentiation of paired conventional moDCs and CXCL4-moDCs on day 2, day 4, and day 6. Conventional moDCs and CXCL4-moDCs were harvested and washed with fresh culture medium (without cytokines) on day 6, counted, and plated O/N for a resting period, unstimulated cells (day 7), cells stimulated with polyI:C for 4 h (day 7 + 4 h) and for 24 h (day 8) were also lysed in RLTplus buffer (Qiagen) containing 1% (v/v) beta-mercaptoethanol (Sigma). In total, we obtained 65 paired samples for RNA sequencing and DNA methylation profiling. DNA and RNA were extracted using the Allprep Universal Kit (Qiagen) following the manufacturer's instructions. For the experimental validation using transfected moDCs and fibroblasts, due to the limiting number of cells, the total RNA was isolated using an RNeasy Micro Kit (Qiagen) according to the manufacturer's instructions. The concentration of DNA and RNA was assessed using the Qubit RNA HS Assay Kit and Qubit dsDNA HS Assay Kit (Life Technologies), respectively, and measured in the Qubit 2.0 fluorimeter (Invitrogen).

### RNA Sequencing

RNA Sequencing (RNA-seq) was performed at the Genomic Facility from the University Medical Center of Utrecht. RNA integrity was first evaluated using a Bioanalyzer (Agilent). RNA-seq library was prepared using 100 ng total RNA using the TruSeq kit (Illumina). Oligo(dT) magnetic beads were used to enrich for messenger RNAs which were then fragmented (about 200 bp). Random hexamer-primers were used to reverse transcribe mRNA into double stranded cDNA, which was then end-repaired followed by addition of 3'-end single nucleotide adenine. Sequencing adaptors were ligated to the resulting cDNA that was subsequently amplified using PCR. Agilent 2,100 Bioanaylzer and the ABI StepOnePlus Real-Time PCR System were used to assess the quality and quantity of RNA-seq libraries. The library products were sequenced on an Illumina NextSeq 500 sequencer using 75bp single-end reads, generating on average 26.2 million clean reads per sample.

### Transcriptional Data Analysis

For each of the 65 transcriptional profiles, reads were aligned using STAR aligner using the default parameters to the 65,217 annotated genes obtained from the GrCh38 (v79) built from the human genome (http://www.ensembl.org). On average 22.5 million uniquely mapped reads were obtained per sample. The read counts per gene were quantified by the Python package HTSeq (31) using annotations from the GrCh38 (v79) built from the human genome (http://www.ensembl.org). Differentially expressed genes (DEGs) were identified by using the DESeq2 (1.8.2) Bioconductor/R package (32) using likelihood ratio test (LRT), and genes with FDR adjusted *p* < 0.05 were considered differentially expressed. Raw count data were transformed to count per million (CPM) for gene expression visualization. Variance stabilizing transformation (VST) was applied to obtain the VSD data for further analysis (32).

### DNA Methylation Profiling

DNA methylation profiling was performed at the GenomeScan (GenomeScan B.V., Leiden, The Netherlands). Genomic DNA was bisulfite-converted using the EZ DNA Methylation Gold Kit (Zymo Research) and used for microarray-based DNA methylation analysis on the HumanMethylation850 BeadChip (Illumina, Inc.), according to the manufacturer's instructions. Beadchip images were scanned on the iScan system and the data quality was assessed using the minfi (version 1.20.2) package (33) using default analysis settings.

### DNA Methylation Data Analysis

Illumina Infinium HumanMethylation850 BeadChip fluorescent data (>850,000 CpG sites) were imported and transformed to methylated (M) and unmethylated (U) signal by minfi package (33). CpG probes were quality-checked and filtered using the following criteria: (i) probes that failed in at least 5% samples were removed, (ii) probes with bead count <3 in at least 5% of samples were removed, (iii) probes targeting SNP sites were removed, and (iv) probes that aligned to multiple locations were removed as described (34). We further removed the probes for the sex chromosomes. One sample (102920-001-17, moDC differentiation sample from donor 4 on day 2) did not pass the quality check and was removed from the subsequent analysis. Approximately 558,000 CpG sites located in six regions (TSS1500, TSS200, 5'UTR, 1stExon, Exon boundaries, and 3'UTR) remained after the quality checks. The intra-array data normalization for the bias introduced by two types of Infinium probes was performed by Beta-mixture quantile normalization (BMIQ) method in ChAMP (version 2.6.0) package (35). The DNA methylation level of each CpG was depicted by the ratio of methylated (M) signal relative to the sum of both methylated and unmethylated (U) signal:

$$\begin{array}{l} \beta =\frac{M}{M+U+100} \end{array}$$

We studied the alterations of DNA methylation considering: (i) individual CpG sites, (ii) region of the CpG site (including 1,500 base pairs before TSS or TSS1500, TSS200, 5'UTR, 1st Exon, Exon boundaries, and 3'UTR), and (iii) proximal genes. To find the differentially methylated CpGs (DMPs) of conventional moDC or of CXCL4-moDC associated with time, a linear regression model with two variables (donor and time) was fitted at each probe. We analyzed DMPs separately for differentiation and stimulation experiments. CpG sites with time-associated FDR corrected *p* < 0.05 were considered DMPs. Similarly, DMPs between conventional moDCs and CXCL4-moDCs were identified using a linear regression model with three variables (donor, time and condition). To obtain region-specific β-values, we calculated the average β-values using all probes that mapped to the same region [including TSS1500, TSS200, 5'UTR, 1stExon, ExonBnd, and 3'UTR (36)] for a given gene. We then applied the same regression models to find differentially methylated regions (DMRs). If any of the regions around the gene were significantly altered, we considered that the gene was differentially methylated (DMGs).

### Multidimensional Scaling (MDS) Analysis

Transcriptional data (VSD) and DNA methylation data (β) were utilized to visualize the differences of cells during differentiation and polyI:C stimulation. The Euclidean distances between samples were calculated based on VSD or β. Multidimensional scaling was performed using these distances in R (cmdscale function from stats package) to project (visualized using ggplot2 package) the high dimensional transcriptional or DNA methylation data onto two dimensions. MDS plots were generated using the DEGs or DMRs.

### Comparison of Gene Expression and DNA Methylation

To compare the relationship between expression and methylation data, we analyzed the two-layered data from genes that were both differentially expressed and methylated. We calculated Spearman correlation coefficients (for Figure 3A, Figures S4B–D) between the expression (VSD) and methylation (β values) data for the genes using the cor function in R. To ensure paired analysis, we removed the corresponding expression profiles for the sample which failed the DNA methylation quality checks. Thus, we performed all correlation-based analysis using 64 pairs of samples. To study the global relationship between gene expression and DNA methylation, contour plots were constructed for paired expression (VSD) and methylation (β values) data using geom_density2d function in R (Figures S4F,G). For Figure S4F we used the paired data from all the genes, while for Figure S4G we used the paired data from all the genes that were both differentially expressed and differentially methylated. We further analyzed the relationship between the paired expression (VSD) and methylation (β values) data using linear regression models and by fitting smoothing curves using generalized additive model (GAM).

### Pathway Enrichment Analysis

Pathway enrichment analysis, for DEGs, DMGs or module genes, was performed using hypergeometric test in ReactomePA package (37). The compareCluster function in the ReactomePA package (with parameters fun = “enrichPathway,” pAdjustMethod = “fdr,” and pvalueCutoff = 0.05) was used to compare and plot the pathways enriched in different sets of genes.

### CIITA-Silencing in Monocytes

Freshly purified monocytes were cultured in medium without antibiotics at a density of 2 × 10^6^ cells/ml. Transfection mix was prepared with 40 nM of Silencer® pre-designed siRNA against human CIITA (targeting exon 3 and 4; siCIITA) or the Silencer^TM^ Negative Control No.1 (siControl) (Life technologies), Lipofectamine 2,000 and Plus Reagent (both from Invitrogen), diluted in Opti-MEM® I Reduced-Serum Medium (Life Technologies). After 5 h, transfected cells were washed with culture medium, and cells were differentiated with IL4 and GM-CSF for 6 days. To analyze viability, transfection efficiency and the effect of transfection on cell phenotype, we harvested cells on day 2, 4, and 6. To access viability cells were incubated with a viability dye (Fixable Viability Dye eFluor780, eBioscience) and analyses were performed by flow cytometry (Figure S8E). To determine the transfection efficiency, cells were transfected also with non-targeting siRNA labeled Cy3, and analyses were performed by flow cytometry (Figure S8F).

### Fibroblast Cultures

Dermal fibroblasts (DF) were isolated from healthy skin biopsies. Skin biopsies were obtained from unused material after cosmetic surgery from anonymous donors who had given prior informed consent to use the biopsies for research. The use of this material is exempted from ethical review processes. DF were isolated using the Whole Skin Dissociation Kit (MiltenyiBiotec) following the manufacturer's instructions, cultured in DMEM medium (Life Technologies) supplemented with 10% (v/v) FBS, and 1% (v/v) antibiotics (used for experiments between passages 4 and 5). Prior to the treatment, DF were cultured O/N with DMEM medium containing 1% FBS. Supernatants collected from conventional moDCs and CXCL4-moDCs stimulated with polyI:C for 24 h were added to the DF for 24 h. Medium and polyI:C were also added to the DF as controls.

### Real-Time Quantitative PCR

Purified RNA was retro-transcribed with iScript Reverse Transcriptase Kit (Bio-Rad). Gene expression was measured by Real-Time quantitative-PCR (RT-qPCR) on the QuantStudio 12k flex system using SybrSelect Mastermix (Life Technologies). The thermocycling conditions were as follow: 95°C for 5 min, followed by 40 cycles of 95°C for 15 s and 60°C for 1 min. To calculate the ratio between the expression of a gene of interest and housekeeping genes (mean between *RPL32* and *RPL13A* for moDCs cultures; RPL13A for the fibroblasts cultures), we used either the 2^−−DCt^ or the 2^−−DDCt^ method. Primer sequences are listed in the online Table S2.

### Cytokine Production Measurement

To validate secreted targets at the protein level, we collected cell-free supernatants after conventional moDC and CXCL4-moDCs differentiation (day 6) and after 24-h stimulation with polyI:C (day 8) from the same 5 HDs that were used for RNA sequencing and DNA methylation profiling. Cytokine measurements were assessed using Luminex assay as previously described (20) at the MultiPlex Core Facility of the Laboratory of Translational Immunology (University Medical Center Utrecht). Data were acquired using Bio-Rad FlexMap3D system and the Xponent 4.2 software, and analyzed using Bio-Plex Manager (version 6.1).

### Flow Cytometry

To measure viability, after moDC differentiation, stimulation or transfection, cells were first incubated with Fixable Viability Dye eFluor780 (eBioscience) in PBS to exclude dead cells. Non-specific Ab binding was prevented by treating the cells and were further treated with 10% (v/v) mouse serum (Fitzgerald). For phenotypic analysis, cells were stained with the following anti-human fluorochrome-conjugated mAbs: CD14 (1:120 dilution; clone M5E2), CD86 (1:80 dilution; clone IT2.2) and CLEC10A (1:50 dilution; H037G3) obtained from BioLegend, CD1a (1:70 dilution; clone HI149) and LAMP1/ CD107a (1:50 dilution; clone H4A3) obtained from BD, or the isotype control-matched Ab. Cells were acquired on the LSR Fortessa (BD) and data was analyzed using the FlowJo software (version 7.6.5; Tree Star. Inc.).

### Western Blot

Conventional moDCs and CXCL4-moDCs after 4 and 6 days of differentiation were washed with PBS and lysed in Laemmli buffer. The same was performed for moDCs differentiated for 6 days after transfection with siControl and siCIITA. Protein concentration was quantified using the Pierce BCA Protein Assay Kit (Thermo Scientific) according to the manufacture's protocol. Equal amounts of protein from different lysates were mixed with loading buffer and boiled at 95°C for 5 min. Next, protein lysates were separated by electrophoresis on a 4–12% Bis-Tris SDS NuPAGE gels (Invitrogen) and transferred to a PVDF membrane (Millipore). After blocking the membranes with Tris-buffered saline (pH 8) containing 0.05% Tween-20 and 4% milk (Bio-Rad) for 1 h at room temperature (RT), the membranes were probed with the antibodies recognizing FN1 (1:500 dilution; Abcam) and tubulin (1:10,000 dilution; Sigma-Aldrich) O/N at 4°C. Afterwards, membranes were washed and incubated for 1 h at RT with the secondary anti-rabbit or anti-mouse antibodies, both HRP-conjugated (Dako). Protein detection was assessed using a ChemiDoc MP System (Bio-Rad). Protein visualization and densitometry analysis of band intensity were performed using the Image Lab software (version 5.1, Bio-Rad). We calculated the ratio between the expression of FN1 and tubulin to determine the relative expression of FN1 in different conditions.

### Confocal Microscopy

As an alternative way to validate the expression and production of FN1, we performed microscopy analyses as described before (20), with minor modifications. For the differentiation of both moDCs, we used Nunc® Lab-Tek® II chamber slides (Thermo Scientific) pre-coated with 0.01% (v/v) poly-L-lysine (Sigma-Aldrich) in sterile water, for 30 min at 37°C. Chamber-wells were washed with PBS and air-dried prior to conventional moDC differentiation. For conventional moDC and CXCL4-moDC differentiation the procedures were the same described above. After differentiation, cells were incubated with fixation/permeabilization solution (eBioscience) supplemented with 5% (v/v) normal goat serum (Cell Signaling) for 30 min at RT, followed by two washes with permeabilization buffer (eBioscience). Cells were incubated for 1 h at RT with primary antibody recognizing FN1 (1:100 dilution; Abcam). After washing twice, cells were incubated with secondary antibody Alexa 594 anti-rabbit (1:800 dilution; Life Technologies) and phalloidin-labeled FITC (1:200 dilution) ENZO) for 1 h in permeabilization buffer, in dark at RT. Cells were washed and incubated with Hoechst 33,342 (1 μM; Invitrogen) for 15 min. Next, cells were washed with permeabilization buffer twice, and at last washed with 1% (w/v) BSA and 0.1% (v/v) sodium azide (NaN_3_; Sigma-Aldrich) in PBS. Mowiol (Sigma-Aldrich) was used to mount the dry slides and coverslips. Image acquisition was performed on a LSM710 (Zeiss) confocal microscope using the Zen2009 (Zeiss Enhanced Navigation) acquisition software. Confocal images were obtained with the objective 63x 1.40 oil and analyzed using the ImageJ software.

### RegEnrich Pipeline

We developed a data driven pipeline (RegEnrich) to integrate CXCL4 specific transcriptional and DNA methylation signatures and to predict the key TFs driving the differential transcriptional profile of CXCL4-moDCs compared to conventional moDCs (Figures 3B–E, 4A–B). RegEnrich pipeline involves three steps: (1) construction of data-driven networks; (2) deducing genes of interest; and (3) enrichment of transcriptional factors or regulators (henceforth called “*TF*”). The aim of RegEnrich pipeline is to rank TFs based on their differential expression and the enrichment of their own downstream targets in a given gene set. RegEnrich pipeline can be made available upon request from the authors and the major steps in the pipeline are shown as following three sections:

### Co-expression/Co-methylation Network Construction

For co-expression network, VSD data of all DEGs were used to construct a co-expression network by R package WGCNA (version 1.51) (38) as described in https://horvath.genetics.ucla.edu/html/CoexpressionNetwork/Rpackages/WGCNA/Tutorials/. Briefly, we used unsigned correlations and a soft thresholding power of 6 to construct networks with scale free topology. We calculated the adjacency matrix which was further used to calculate Topological overlap matrix (TOM) to identify modules of co-expressed genes. Modules were identified using cutreeDynamic function with the minimum module size of 30. Modules were further merged if the Pearson correlation of their eigengene was <0.25. Using this methodology, we obtained 27 co-expression modules (Figure S5A). Nodes (genes) and edges (connections of genes) in each module were exported by exportNetworkToCytoscape function (threshold ≥ 0.02).

To build co-methylation network, we first assigned a unique β value to a given gene of a sample by setting priority to four regions: TSS200>TSS1500>5'UTR>1stExon as described in Jiao et al. (36). If for a gene TSS200 region is differentially methylated (DM), we considered the β value of TSS200 as the methylation level of this gene. Similarly, for a gene without DM TSS200 but with DM TSS1500, β value of TSS1500 region was used, and so on. Then these regions, representing corresponding genes, were used to build co-methylation networks using the methodology described for the co-expression network. To achieve topological scale-free networks, standard parameters were set to a soft thresholding power of 12, “unsigned” network, minimum module size of 30, merged module threshold <0.25, and an exporting network threshold of 0.02. In total, 10 modules were reserved in the end (Figure S5B).

### Gene Regulatory Network (GRN) Construction Based on Random Forest (RF) Algorithm

To obtain potential transcription (co-)factor/regulators (TF) for each gene in a data-driven manner, we constructed a TF-target GRN using random forest machine learning algorithm [modified from (39, 40) and is part of RegEnrich package developed by us; see below]. This TF-target GRN is a directed network of two types of components: (a) TFs and (b) their potential targets. Here targets might not necessarily be direct downstream targets that the TFs might bind to, rather the genes that are inferred to be directly/indirectly regulated by the TFs based on the transcriptional data. The construction of TF-target GRN consisted of four steps. First, the VST normalized data from 17,709 DEGs (same genes in co-expression network), including 1,172 differentially expressed TFs (Table S9), were selected for the analyses. Second, we removed all the target genes that are expressed in <10 samples. Third, for every target gene, a random forest model was built to predict its expression based on TF expression (the parameters are: K = “sqrt,” nb.trees = 1,000, importance.measure = “IncNodePurity”). As a last step, models with low performance (MSE < 0.5) were removed to achieve a robust TF-target GRN.

### TF Enrichment Analysis

In this study, TF enrichment analyses were performed on two data-driven networks, a co-expression network and a GRN network. For the co-expression network, we ranked the edges between TFs and their potential targets based on the edge weight. Top 5% edges were then selected and were considered for further analyses. This resulted in 1,037,689 TF-target connections. Similarly, for the GRN network, we used top 5% of edges (688,559 TF-target connections). One-tailed hypergeometric test was used to calculate the enrichment *p*-values (*p*_*E*_) for each TF in a given set of genes (here genes differential between CXCL4-moDCs and conventional moDCs). Those TFs that exhibited significant differential expression (*p*_*D*_ < 0.05) and had significant enrichment (*p*_*E*_ < 0.05) were considered as key TFs. In other words, TFs that were differentially expressed along with their own targets were considered to be enriched in a given gene set. The overall scores of TFs were calculated by:

$$\begin{array}{l} score=norm\left(-\log\left(p_{E}\right)\right)+norm\left(-\log\left(p_{D}\right)\right),wherenorm\left(x\right)\\ \;=\frac{x-\min\left(x\right)}{\max\left(x\right)-\min\left(x\right)}. \end{array}$$

Cytoscape 3.4 (www.cytoscape.org) was used to visualize the networks. In TF-TF networks, we only plot the edges connecting the enriched key TFs in both co-expression and GRN network. For better visualization, only TFs with |log_2_(*fold change*)| > 0.6 were shown (Figures 4A,B, Figures S7A,B).

### Visualization of Co-expression and Co-methylation Network Integration

Spearman correlation coefficients were calculated using the co-expression and co-methylation module eigengenes to integrate the two networks (Figure S5C). We calculated the number of genes shared between co-expression and co-methylation modules and two-tailed fisher exact test was used to evaluate the significance of each overlap (Figure 3C). Pearson correlation coefficients were used to relate gene modules to sample traits i.e., CXCL4^+/−^, time and polyI:C^+/−^ (Figure 3C).

### Statistical Analyses

Unless stated all statistical analyses were performed using R. The detailed analysis of RNAseq data and DNA methylation data generated in this study were described in the aforementioned sections. For RT-qPCR data (Figures 4D, 5F,I, 8C, 9A–C, Figure S7D), Luminex data (Figures 1I,K, 5B, Figures S1E, S2E), module membership data (Figures 3D,E), Pearson chi-square test was first used to verify the hypothesis of normality of the differences of paired samples. All Luminex data, module membership data, and RT-qPCR data (except HLA-DRA & IRF8 in Figure 4D; FN1 in Figure 5F; FLT1, CD86, CCL3, CHI3L1, and LGALS9 in Figure S7D; IL1RN in Figure S8; COL4A2 & IL6 in Figure S9C) are normally distributed (*p* > 0.05). Paired two-sided Student's *t*-test was used to compare the mean between two groups for these data; the variances of two groups are not treated equal and the Welch approximation to the degrees of freedom is used. For those exceptions, two-sided Wilcoxon signed rank sum test was used. The null hypothesis assumed that there is no difference between the means/signed rank of the two groups. The numbers of samples plotted in each figure were detailed in the corresponding figure legend. Unless stated, *p* < 0.05 were considered as statistically significant.

## Results

### CXCL4 Drastically Impacts Monocyte Differentiation

To study the role of CXCL4 on the possible imprinting of immune cells toward fibrosis, we examined the effects of CXCL4 on the differentiation of monocyte-derived dendritic cells (moDCs) (20). We cultured monocytes obtained from five healthy donors in the presence of IL-4 and GM-CSF to differentiate them in the absence (conventional moDCs) and presence of CXCL4 (“CXCL4-moDCs”). To systematically study the effects of CXCL4 on the trajectory of monocyte differentiation into moDCs, we obtained longitudinal transcriptional (RNA-seq) profiles at days 0 (monocytes), 2, 4, and 6. To examine the effects of CXCL4 on moDC maturation, we stimulated the cells on day 7 with the toll-like receptor 3 ligand polyI:C and obtained transcriptional profiles before stimulation (day 7), 4 h (day 7 + 4 h) and 24 h (day 8) after stimulation (Figure 1A).

The differentiation of moDCs from monocytes was accompanied by extensive transcriptional changes, as 13,192 genes underwent significant (likelihood ratio test; FDR corrected *p* ≤ 0.05) alterations in their expression levels (Figure 1B). Nearly half of the differentially expressed genes (6,350) were upregulated. CXCL4-moDCs also underwent widespread transcriptional changes, as 13,110 genes were differentially expressed compared to monocytes, nearly half of those were found to be upregulated (Figure 1B, Figure S1). Remarkably, most of this transcriptional shift happens between day 0 and day 2, in both conventional moDCs and CXCL4-moDCs (Figure 1D). Genes characteristic of monocyte differentiation (such as *CD14, CD163, TLR2, TLR4*, and *TLR7*) (41) and cell adhesion molecules (including *LGALS2, LGALS9*, and *ICAM2*) were down-regulated in both conventional moDCs and CXCL4-moDCs on day 2 (Table S3). After day 2, cells continued to differentiate, as evidenced by their shifting transcriptional profiles (Figure 1D). Genes encoding pattern recognition proteins MRC1, MRC2, growth factors such as CSF1, and the chemokine receptors CCR1, CCR5, and CCR7, were upregulated over time in both conventional moDCs and CXCL4-moDCs (Table S3). Together these results indicate that the differentiation of monocytes (with or without CXCL4) leads to massive transcriptional changes as reported by several previous studies (42, 43).

To elicit the transcriptional signature unique to CXCL4 exposure, we compared differentiating CXCL4-moDCs with conventional moDCs from day 2 to day 6 and found differential expression of 5,775 genes (likelihood ratio test, FDR corrected *p* ≤ 0.05; Figure 1B, Figure S1, and Tables S1 and S3). CXCL4-moDCs follow a distinct molecular differentiation trajectory that progressively diverges from conventional moDCs (Figure 1D, right panel). The CXCL4 signature genes belong to several crucial innate immune system pathways including cytokine signaling, interferon signaling, and antigen processing and presentation (Figure S1B). For instance, CXCL4-moDCs, in the absence of further stimulation, up-regulate expression of several inflammatory molecules such as *CTSL, FLT1, CD86, LAMP1, CHI3L1*, and down-regulate signaling receptors such as *CLEC10A, IL1R1, IL1R2* compared to moDCs (Figures 1G–I and Figures S1A,C–E). We had previously shown (20) that CD86 was upregulated and CD1a was downregulated in CXCL4-moDCs compared to conventional moDCs. These results were consistent in our current study (Figure S1D). Other pathways such as transmembrane transport of small molecules, ubiquitin-dependent degradation of cyclin D, and innate immune system as a whole are strongly perturbed by CXCL4 during the differentiation of moDCs. Strikingly, CXCL4 exposure also leads to dramatic changes in expression of genes regulating metabolism and transcription (Figure S1B), reminiscent of changes previously observed in myeloid cells undergoing immune training (44). Thus, CXCL4 orchestrates a differentiation process dramatically different than that of the conventional moDCs.

### Mature CXCL4-moDCs Are Functionally Distinct From Conventional moDCs

To study the effects of CXCL4 on moDC maturation, we stimulated the cells with polyI:C on day 7. This perturbed the expression of 8,949 and 7,767 genes in CXCL4-moDCs and conventional moDCs, respectively, compared to the day 7 transcriptional profiles of their unstimulated counterparts (Figures 1C,E, left and middle panels). Two thousand, three hundred ninety-seven genes responded differently to polyI:C stimulation in CXCL4-moDCs compared to conventional moDCs (Figure 1C, Figure S2). Several pathways involved in inflammatory responses such as TLR signaling, interferon signaling, and cytokine signaling, were significantly upregulated in both stimulated CXCL4-moDCs and stimulated conventional moDCs compared to the correspondingly unstimulated cells (Figure S2B). Confirming our previous findings (20), these transcriptional changes were followed by increased production of pro-inflammatory mediators such as IL-1β, IL-6, IL-12, IL-23, IL-27, TNF, and CCL22, and down-regulation of immune-suppressive mediator CCL18 (validated using Luminex assays; see Figures 1J,K, Figures S2A,C–E). Pathways involved in cellular adhesion, integrin signaling, ECM organization, and collagen formation, among others, were upregulated in CXCL4-moDCs upon polyI:C stimulation compared to stimulated conventional moDCs (Figure S2B), indicating that CXCL4 exposure may induce a pro-inflammatory and pro-fibrotic phenotype. Because most of the altered genes were already differentially expressed in immature CXCL4-moDCs (Figures 1E,F, Figure S2C), the unique molecular program induced by CXCL4 is suggestive of genetic imprinting.

### CXCL4 Alters Epigenetic Imprinting During Differentiation but Not Maturation of moDCs

**Figure 2:**
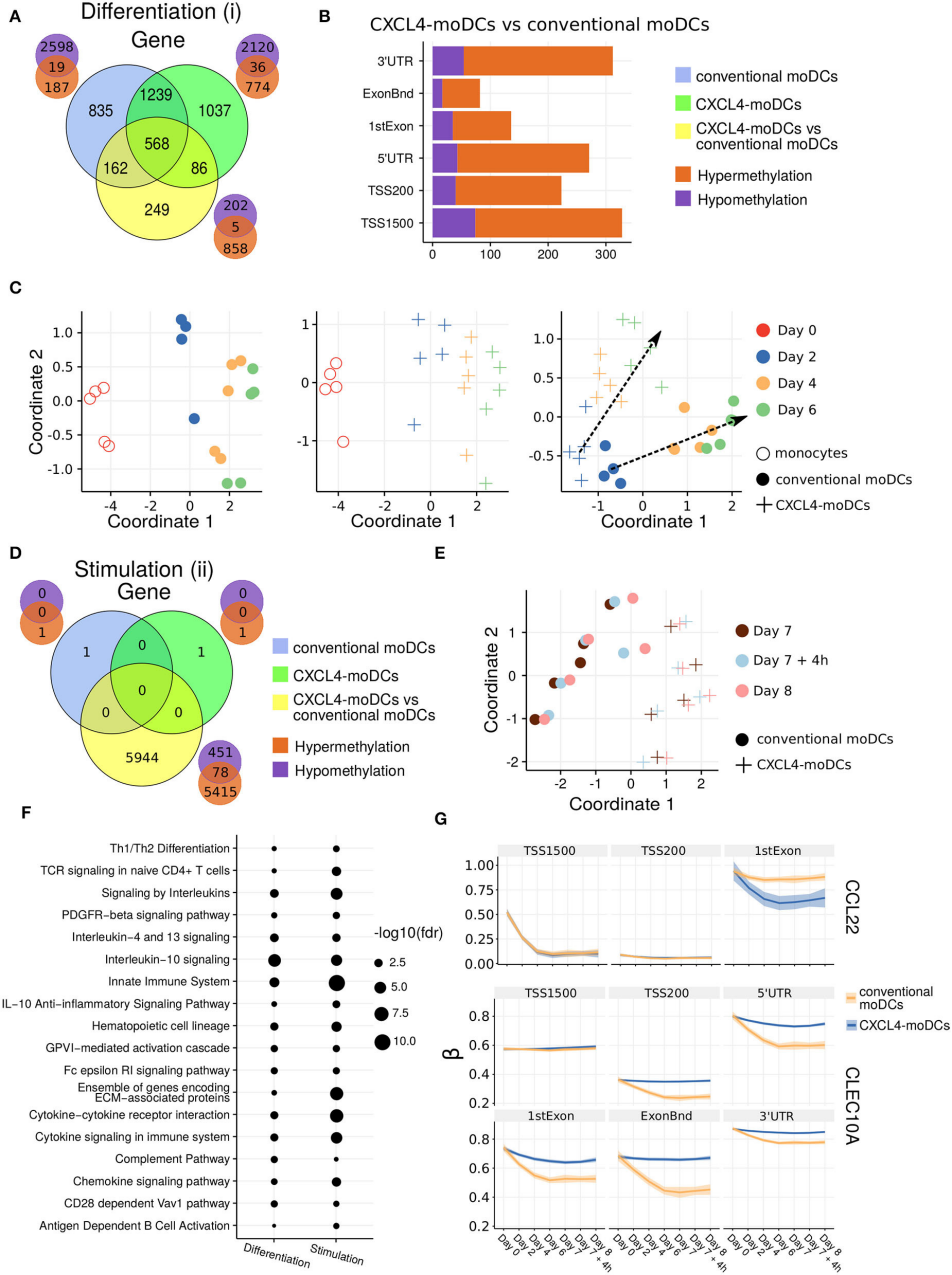
DNA methylation analysis of CXCL4-moDCs and conventional moDCs. **(A)** Overlap between differentially methylated genes (DMGs) found during differentiation similar to Figure 1B. A gene is considered differentially methylated if any region on the gene is differentially methylated. Smaller Venn diagram graphs display the overlap of hyper-methylated (orange) and hypo-methylated (purple) genes for each comparison. Note some genes are classified as both hyper-methylated and hypo-methylated based on different regions. **(B)** Distribution of differentially methylated regions (1,500 and 200 base pairs upstream of the transcription start site (TSS), 5' untranslated region (UTR), 1st exon, other exons (ExonBnd) and 3' UTR) between CXCL4-moDCs and conventional moDCs during differentiation. **(C)** MDS analysis using DMRs, similar to Figure 1D. **(D)** Overlap between DMGs found during stimulation similar to Figure 1C. **(E)** MDS analysis using all DMRs between CXCL4-moDCs and conventional moDCs during stimulation. **(F)** Top enriched pathways from DMGs between CXCL4-moDCs and conventional moDCs during differentiation and stimulation. **(G)** DNA methylation β values (see Methods) of CCL22 and CLEC10A. Lines represent mean β values and shading represents 95% confidence interval.

To comprehensively examine whether CXCL4 signaling might alter moDC phenotype via epigenetic modifications (43, 45, 46), we studied genome wide alterations in DNA methylation. Similar to the transcriptome analysis, we found that a large number of genes, regions and sites were differentially methylated between monocytes and differentiating moDCs and CXCL4-moDCs (Figure 2A, Figures S3,4). Interestingly, most of the differentially methylated genes were hypomethylated compared to monocytes (2,617 in conventional moDCs and 2,156 in CXCL4-moDCs) (Figures 2A,B, Figures S3A, C, and Table S4).

To discern the epigenetic footprint of CXCL4 during differentiation, we compared the methylome profiles of differentiating CXCL4-moDCs with differentiating conventional moDCs (from day 2 to day 6). CXCL4 exposure led to substantial changes in the DC methylome as 1,065 genes were differentially methylated between CXCL4-moDCs and conventional moDCs (Figure 2A, Figure S3). Most of the differentially methylated genes were hypermethylated in CXCL4-moDCs. The hypermethylation was not restricted to promoter regions, indicating that CXCL4 influences chromatin accessibility at a more global level (Figure 2B). Alterations in DNA methyltransferases and DNA demethylases are known to cause global hypermethylation, which have been implicated in SSc pathogenesis previously (47, 48). Interestingly, we found transcriptional upregulation of DNA methyltransferases (such as DNMT3A) and downregulation of DNA demethylases (TET2 and TET3) that together can cause global hypermethylation in CXCL4-moDCs (Figure S4A). As in the transcriptional analysis, CXCL4-moDCs progressively diverge from moDCs (Figure 2C, right panel). This progressive and temporal divergence of DNA methylation patterns caused by CXCL4 alters several crucial innate immune system pathways including cytokine signaling, co-stimulatory molecules, and ECM organization (Figure 2F). Thus, we provide potential mechanistic evidence that CXCL4 programs a pro-inflammatory and pro-fibrotic phenotype via epigenetic imprinting that corroborates the transcriptional results (Figures S2F, S3E).

We also studied the role of epigenetic remodeling in mature moDCs. Surprisingly, stimulation of conventional moDCs and CXCL4-moDCs with polyI:C on day 7 hardly affected the DNA methylation (Figures 2D,G, Figures S3B,D), an observation confirmed by multivariate analysis as the samples did not exhibit any temporal clustering (Figure 2E). Thus, the altered functional responses exhibited by CXCL4-moDCs were epigenetically imprinted during differentiation rather than maturation (Figure S3).

One of the aims of this study was to understand the role of CXCL4 in regulation of inflammatory genes. We found that CXCL4 modulates the methylation and expression profiles of several inflammatory genes. For example, the expression of CCL22, an important gene involved in systemic sclerosis (49), dramatically increased during the differentiation of CXCL4-moDCs. Interestingly, TSS1500 region of CCL22 is observed substantially demethylated and 1stExon region of CCL22 is significantly hypomethylated in CXCL4-moDCs compared to conventional moDCs during differentiation (Figure 2G). Further upon polyI:C stimulation, the methylation status of these regions persists (Figure 2G). Similarly, in Figure 1H, Figure S1C, we showed that the gene and protein expression of CLEC10A, an important gene in antigen uptake and DC maturation, decreased in CXCL4-moDCs compared to conventional moDCs. We found that CLEC10A was hypermethylated in differentiating CXCL4-moDCs compared to conventional moDCs. Thus, CXCL4 modulates the expression of several inflammatory genes potentially by directly/indirectly inducing changes in the DNA methylation profiles of differentiating moDCs.

### Gene Regulatory Network Driving the CXCL4-Specific Transcriptome

**Figure 3:**
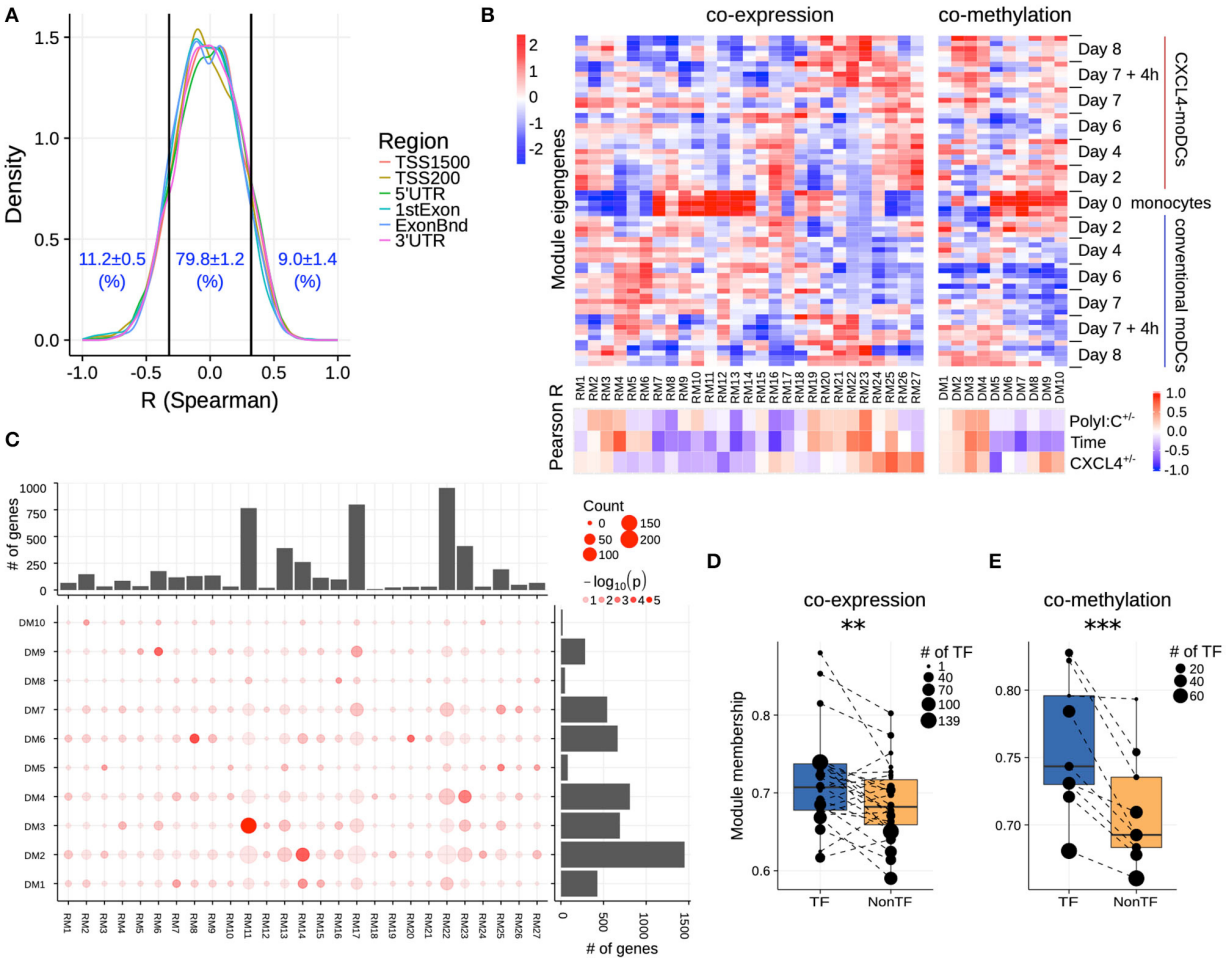
Co-expression and co-methylation networks. **(A)** Distribution of spearman correlation coefficients (R) between β values of each region and the corresponding gene expression for all genes that are differentially expressed and methylated. The cutoffs (two vertical lines at *R* = ±0.32) indicate significant correlation coefficients (*p* < 0.01). **(B)** The top heatmap shows expression/methylation eigengenes of co-expression (left) and co-methylation (right) modules. The bottom heatmap shows the Pearson correlation coefficients between sample traits (i.e., CXCL4^+/−^, time and polyI:C^+/−^), and co-expression (left) and co-methylation (right) module eigengenes. **(C)** Concordance of co-expression and co-methylation modules. The bottom left graph shows the number (circle size) and significance (color, *p*-value calculated by Fisher's exact test) of overlapping genes between co-expression and co-methylation modules. The bar plots show the total number of genes in the co-expression (top) or co-methylation (right) module. Module membership comparisons of transcriptional regulators (TF) and other genes (NonTF) in **(D)** co-expression and **(E)** co-methylation network. Each dot represents a module and the size denotes the number of TFs in the corresponding module. Modules that do not contain TFs were excluded in these analyses. **P < 0.01, ***P < 0.001, paired two-sided Student's *t*-test.

Since CXCL4 exposure caused massive alterations in both DNA methylation and transcriptional factors, we next studied the regulatory mechanisms behind the CXCL4 signature. We first assessed the concordance of DNA methylation and mRNA expression and found that the changes in DNA methylation did not correlate with the changes in corresponding gene's expression for majority of the CXCL4 signature genes (Figure 3A, Figures S4B–E). For example, about 79.8% of these genes did not show significant correlation (*p* > 0.01; 65.5% of these genes did not show significant correlation if the cutoff is *p* > 0.05) between mRNA expression and DNA methylation of the corresponding regions (Figure 3A). We further checked if levels of DNA methylation reflect upon the overall gene expression levels, rather than their differential expression. We indeed found that levels of DNA methylation play a role in overall gene expression levels (Figures S4F,G). Since we did not find significant correlations between transcriptional and DNA methylation changes for a large fraction of genes, we further focused on other aspects that may contribute toward regulation of CXCL4-moDCs.

Genes rarely work in isolation, and their expression is typically regulated via a complex molecular network (50, 51). To systematically identify the underlying complexity and inter-connectivity of molecular changes caused by CXCL4, we developed a new methodology (RegEnrich) to integrate the transcriptional and epigenetic layers and identify the important transcription factors modulated by CXCL4 (see Methods). Using RegEnrich, we first constructed weighted gene correlation networks, which allowed us to cluster genes into distinct modules (or sets of genes) based upon either their co-expression or co-methylation patterns (Figures S5A,B) (38, 52). Modular analysis segregated the differentially expressed genes into 27 modules, each exhibiting a distinct co-expression pattern (Figures 3B,C, Figure S5C). There were modules specific for monocytes, such as RM11, which involved in pathways in innate immune system, Cell surface interactions at the vascular wall (Figure 3B, Figures S6A,B). Also, modules specific for PolyI:C stimulation were found, such as RM22, which is related to Innate Immune System, cytokine signaling in Immune system (Figure 3B, Figures S6A,B), etc. Of note, some CXCL4-moDCs-specific modules were also found to be related to CXCL4-moDC phenotypes. For example, as one of CXCL4-moDCs-specific modules, module RM25 contained genes belonging to ECM organization, ion channel transport, IFNα signaling and metabolic pathways (Figure 3B, Figure S6B). Similarly, modular analysis segregated all differentially methylated genes into 10 distinct co-methylation modules (Figure 3B). Monocyte-specific module (DM7) and CXCL4-moDC-specific modules (DM5 and DM9) were also observed. And the CXCL4-moDC-specific modules contained genes belonging to transcriptional and translational pathways, antigen presentation pathways, and the innate immune system (Figure 3B, Figure S6C; Table S8). However, we did not find much overlap between the CXCL4-moDC-specific co-expression and co-methylation modules, except those module pairs which eigengenes are not correlated, such as module pair DM3-RM21 (Figure 3C). In addition to from single gene perspective, here, we observed from an integrated network perspective that DNA methylation maybe only partially influences the transcriptional changes of CXCL4 signature genes, suggesting that other factors might exist to induce such massive transcriptional changes.

**Figure 4:**
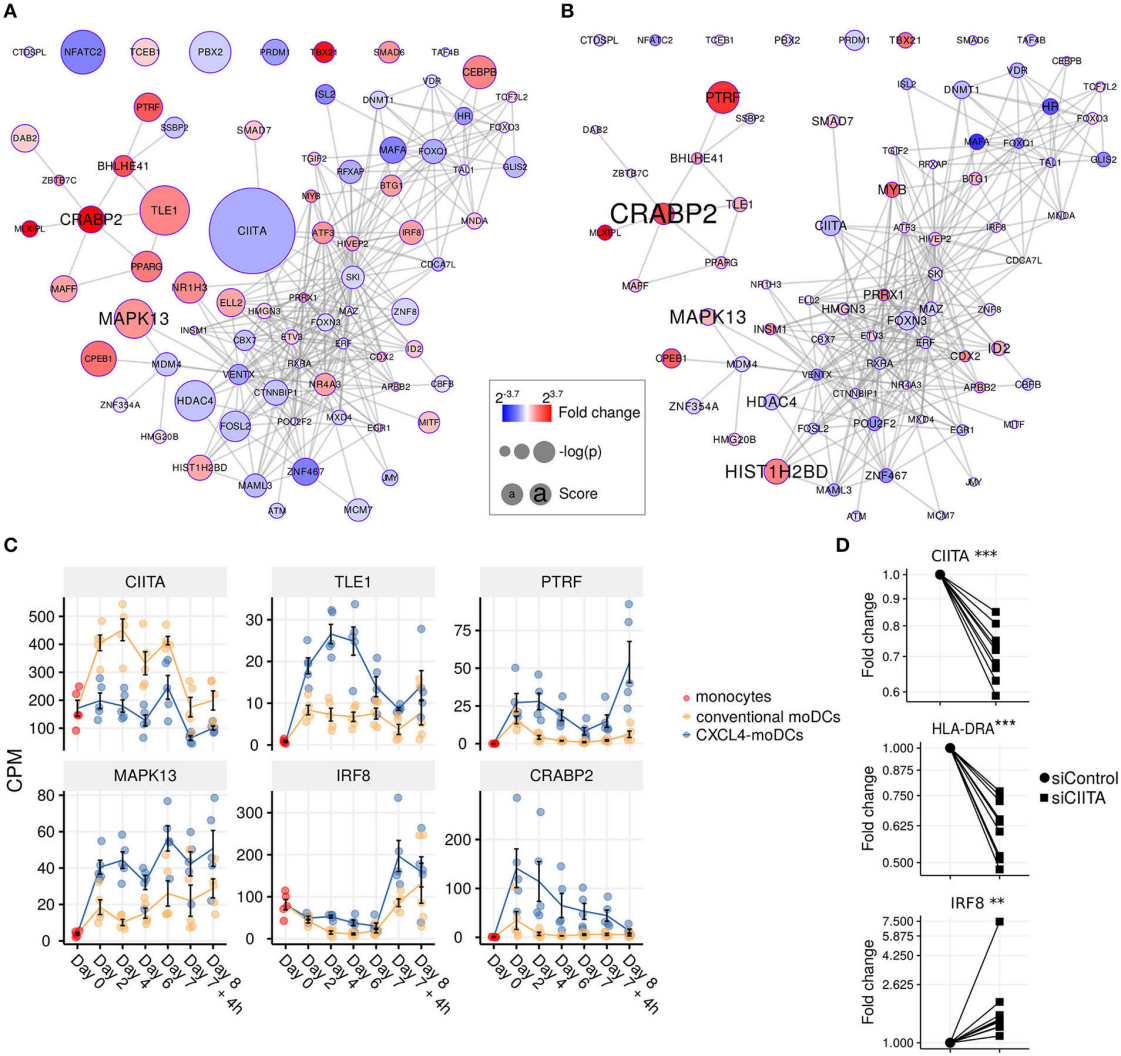
Transcription regulator enrichment highlights key TF candidates. Co-expression based key TF network during: **(A)** differentiation and **(B)** stimulation. Colors indicate fold change between CXCL4-moDCs and conventional moDCs. Red represents upregulation and blue represents downregulation. Circle size indicates –log_10_(p) for each comparison, where p is the *p*-value calculated during differential expression analysis; text size shows the RegEnrich score (see Methods). **(C)** Expression profile (mean±SEM) of key regulators. **(D)** Expression of *CIITA, HLADRA*, and *IRF8* on day 6 measured by qPCR in moDCs obtained from monocytes transfected with silencer negative control siRNA (siControl) or silencer CIITA siRNA (siCIITA). qPCR data were normalized using mean expression of *RPL32* and *RPL13A*. Fold change in y-axis (log2 scaled) is relative to the value obtained for siControl for each donor. Lines connect individual donors. *P < 0.05, ***P < 0.001, paired two-sided Student's *t*-test or two-sided Wilcoxon signed rank sum test (see Statistical Analyses in Materials and Methods).

To test whether transcription regulators are the central players (hubs) in our networks (50, 51), we calculated module memberships as a measure to determine the importance of a gene in a given module (38, 52). Interestingly for both co-expression and co-methylation modules, we found that the transcription regulators typically exhibited higher module membership than the other genes (Figures 3D,E and Figure S5D). That transcription regulators are typically the hubs in our networks highlights their crucial regulatory function in modulating the expression dynamics of their downstream target genes. Thus, alterations in the expression and activity of a few key transcription regulators can potentially precipitate the large phenotypic differences observed between moDCs and CXCL4-moDCs. Using RegEnrich, we ranked the transcription regulators most prominently dysregulated between CXCL4-moDCs and conventional moDCs during differentiation (Figure 4A), and post stimulation (Figure 4B). Using these gene regulatory networks, we predicted that key transcription regulators such as *CIITA, TLE1, PTRF, MAPK13, CRABP2, IRF8*, directly or indirectly regulate a large number of the CXCL4 signature genes, including pro-inflammatory and ECM pathway genes (Figures 4A,B). We confirmed that these key transcription regulators were significant for CXCL4 signature genes using an independent approach: random forest-based gene regulatory networks (see Methods, and Figures S7A,B). Together, the data-driven gene regulatory networks identified a potential mechanistic link between CXCL4, inflammation and ECM modeling in moDCs.

### CIITA Is a Key Target of CXCL4 Signaling

We found that the key transcriptional regulatory proteins exhibit different mRNA expression patterns over time. For example, *TLE1, PTRF*, and *CRABP2* were expressed at low levels in monocytes but were upregulated during the differentiation of both conventional moDCs and CXCL4-moDCs (Figure 4C). However, these genes exhibited persistently higher expression in CXCL4-moDCs during both differentiation and following polyI:C stimulation (Figure 4C). Another example is the interferon regulatory factor 8 (IRF8), a transcription factor typically associated with pro-inflammatory gene expression in monocytic lineages, which is markedly upregulated in immature CXCL4-moDCs compared to conventional moDCs (Figure 4C). Class II MHC transactivator (CIITA), a transcription co-factor associated with regulation of MHC class II gene expression, was the most significantly down-regulated regulator in CXCL4-moDCs (Figure 4C).

To validate the inter-connectivity of important regulators inferred from the gene regulatory network, we performed siRNA-mediated knockdown to silence CIITA expression. At day 6 following introduction of siRNA, monocyte-derived cells remained viable, and displayed the anticipated phenotype: CIITA-silencing down-regulated expression of both CD74 and HLA-DR (Figure 4D, Figures S7C,D) (53). While IRF8 has not been reported to be regulated by CIITA, our gene regulatory networks predicted direct or indirect regulatory interactions between CIITA and IRF8. Silencing of CIITA led to upregulation of IRF8, mimicking the effects of CXCL4 and validating the prediction of our gene regulatory networks (Figure 4D, Figures S7C,D). Hence using our gene regulatory networks, we have elucidated novel gene regulatory interactions in moDCs and found that CXCL4 alters the molecular signature changes of moDCs by modulating this network that includes the key transcription regulator CIITA.

### CXCL4 Induces Fibrotic Pathways in moDCs Mediated via Epigenetic Imprinting and CIITA

**Figure 5:**
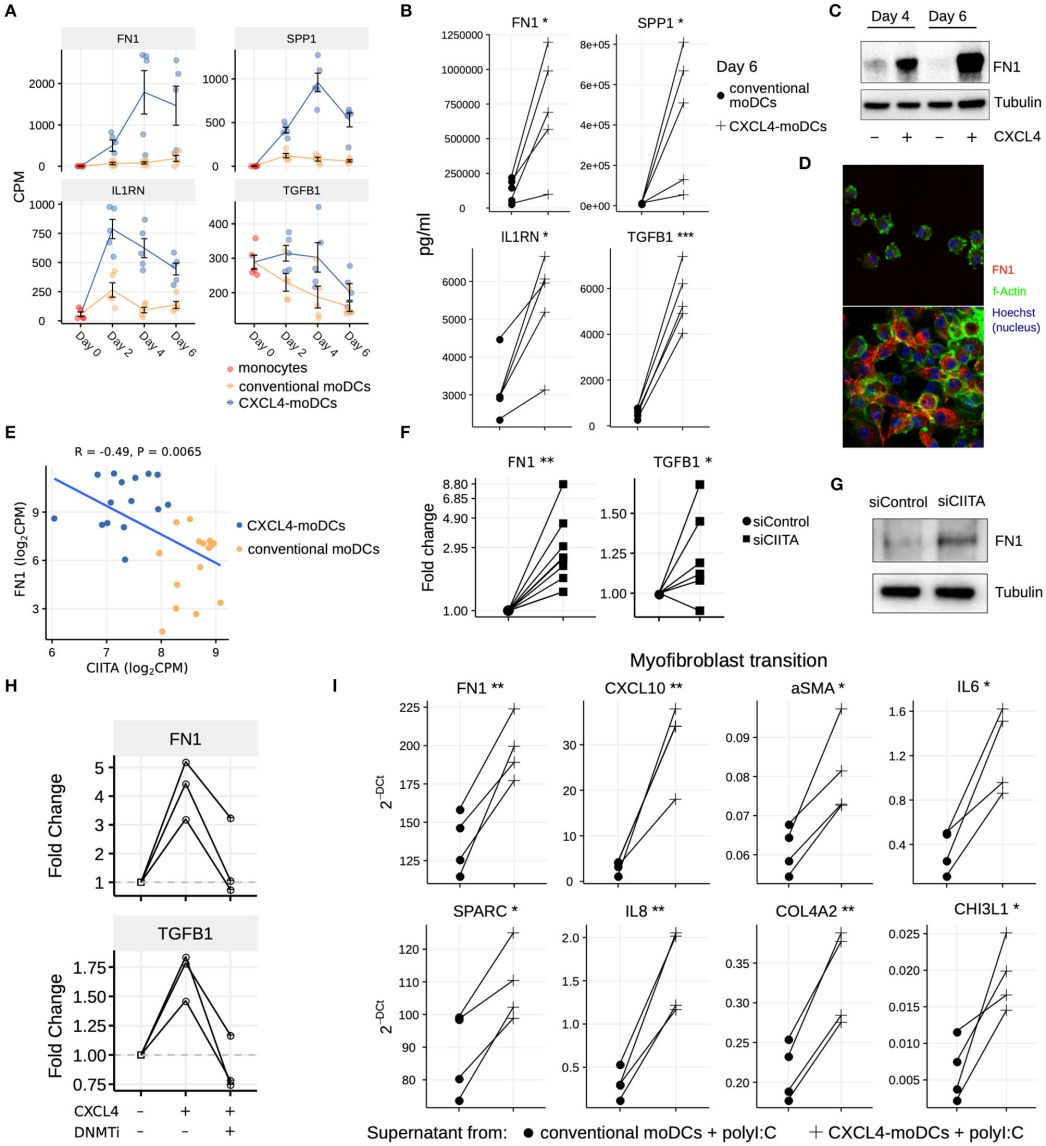
CXCL4 induces production of ECM components in moDCs and fibroblasts. **(A)** Expression of genes implicated in ECM remodeling (mean±SEM). **(B)** Validation (luminex) of ECM protein production in CXCL4-moDCs and conventional moDCs on day 6. **(C)** Fibronectin (FN1) expression (tubulin as loading control) determined using Western blot on days 4 and 6 (representative of 5 independent experiments). **(D)** Fibronectin (red) synthesis determined using confocal imaging on day 6 (green: f-actin; and blue: nucleus staining using Hoechst). **(E)** Pearson correlation between gene expression of *CIITA* and *FN1* during differentiation (i.e., on day 2, 4, and 6). **(F)** FN1 and TGFB1 expression measured by qPCR and **(G)** FN1 expression measured by western blot on day 6 moDCs obtained from monocytes transfected with siControl and siCIITA (see Figure S8D). **(H)** FN1 and TGFB1 expression measured by qPCR on day 3 in conventional moDCs, CXCL4-moDCs and CXCL4-moDCs exposed to DNMT inhibitor (100 nM 5-Aza-2′-deoxycytidine). **(I)** Expression of ECM genes measured using qPCR in healthy dermal fibroblasts (one representative donor; for others see Figure S9) co-cultured with supernatants from CXCL4-moDCs and moDCs that were stimulated for 24 h with polyI:C. qPCR data were normalized using mean expression of *RPL32* and *RPL13A*. In panels **(B,F,H,I)** lines connect individual donors. *P < 0.05; **P < 0.01, ***P < 0.001, paired two-sided Student's *t*-test or two-sided Wilcoxon signed rank sum test (see Statistical Analyses in Materials and Methods).

Our data-driven methodology allowed us to identify several novel regulators and pathways that are differentially regulated due to CXCL4 during moDC differentiation (Figures 2F, 4A–C and Table S7). As a result, we observed that even unstimulated CXCL4-moDCs exhibit a pro-fibrotic phenotype, as characterized by the increased gene and protein expression of several crucial molecules involved in the synthesis and/or degradation of extracellular matrix (ECM), including FN1, SPP1, IL1RN and TGFB1 (for transcriptional changes see Figure 5A, Figures S8A,B; for protein validations see Figures 5B–D). Importantly, silencing CIITA mimicked the effects of CXCL4 leading to upregulation of FN1 along with other molecules involved in ECM remodeling validating the relevance of this network in the pro-fibrotic cascade (Figures 5E–G, Figures S8C,D). Since in CXCL4-moDCs we found that majority of the differential genes were hypermethylated (Figures 2A,B) and that ECM-related genes were up-regulated (including FN1 and TGFB1; see Figures 5A,B), we next examined whether modulating DNA methylation affects FN1 and TGFB1 expression. In line with our hypothesis, inhibition of DNMTs using 100nM 5-Aza-2′-deoxycytidine restored the expression of FN1 and TGFB1 which were upregulated by CXCL4 in moDCs (Figure 5H), suggesting that CXCL4 associated epigenetic imprinting also plays a role in promoting expression of pro-fibrotic genes. Although our data provides unprecedented data for the implication of CXCL4 in fibrogenesis in CXCL4-moDCs, we next examined the possible implication of these CXCL4-moDCs on fibroblast behavior. By culturing fibroblasts with the supernatant of CXCL4-moDCs stimulated with polyI:C, we demonstrate that these fibroblasts expressed markedly higher levels of inflammatory mediators associated with fibrosis and above all, myofibroblast transition, considered indispensable for fibrosis, compared to conventional moDCs stimulated with the same TLR3 ligand (Figure 5I, Figure S9). Together, this data demonstrates that CXCL4 alters the differentiation of moDCs into cells that drive fibrogenesis both directly and, indirectly potentially via TLR3 activation of myofibroblasts.

## Discussion

Although the role of inflammation in fibrosis is increasingly recognized, the underlying molecular links between these processes remain elusive and their identification is paramount for the development of medicines to not only halt progression but prevent fibrosis. Using whole genome transcriptional and epigenetic profiling, we find that CXCL4 drives the development of a pro-inflammatory and pro-fibrotic phenotype in moDCs, characterized by the excessive production of ECM components and capacity to promote myofibroblast differentiation. As these are two key mechanisms contributing to tissue fibrogenesis, our study introduces a novel concept that CXCL4-induced inflammatory DCs, here modeled by moDCs, constitute the driving force behind both the initiation and progression of fibrosis in diseases where CXCL4 levels are increased such as SSc.

TGF-β is considered a key regulator during fibrosis in physiological and pathological conditions (54). For instance, TGF-β drives mesenchymal responses during wound healing, where its transiently increased expression promotes myofibroblast transition. However, the initial stage of wound healing is the formation of a platelet plug, followed by monocyte recruitment. After this primarily inflammatory phase, a switch to resolution occurs that is accompanied by tissue repair and fibrosis (55). Platelets, crucial players in the pathogenesis of several diseases including SSc, contain large amounts of CXCL4 (5, 7, 56). Activation of platelets early on in the wound healing process is likely to precede the synthesis and secretion of TGF-β. Notably, CXCL4 was found to play an important role in lung inflammation and tissue damage (57), and has been identified as a biomarker for early rheumatoid arthritis where it was co-localized with inflammatory cells and platelets in synovial tissue (23). In contrast to other inflammatory mediators that appear at later stages of disease, CXCL4 levels are also increased in patients at risk for SSc, a disease in which clinical inflammation precedes fibrosis by years (7). Together, these observations indicate an early role for CXCL4 in inflammatory and subsequent fibrotic processes, placing CXCL4 upstream of TGF-β. This possibility is further substantiated by our finding that CXCL4 clearly induces TGF-β RNA and protein expression (Figures 5A,B).

Multiple studies provide compelling evidence for the presence of inappropriately activated and/or trained innate immunity in patients with inflammatory diseases. Recently, several crucial studies have highlighted the molecular basis, relevance and pathological consequences of innate immunity trained by various exogenous ligands and endogenous ligands the latter contributing to atherosclerosis and gout (58–61). Following a seminal study which observed enhanced collagen synthesis in SSc patient skin fibroblasts compared to those of healthy control (62), this phenomenon was observed in SSc patient DCs, which had potentiated responses to various TLR agonists (7, 63).

In this study, we profiled longitudinal transcriptome and DNA methylome during moDC differentiation with/without the presence of CXCL4 and followed PolyI:C stimulation. Although we did find some genes with negative correlation between there gene expression and DNA methylation, this negative correlation pattern does not generally apply to the majority of genes. From the co-expression and co-methylation network perspective, it is still challenging to interpret as the DNA methylation play a very important role in regulating the gene expression. A recent study might support our finding, which suggested that DNA methylation would rather play a role in fine tuning gene expression regulation (64). And then by applying RegEnrich, we predicted a key regulator network which potentially play important roles in CXCL4-moDCs phenotyping. As a limitation of this study, the mechanic findings in this study are based on *in vitro* experiments, further *in vitro* validations need to be carried out. For example, CXCL4-moDCs can be transplanted to mice to study the potentiation of pro-inflammatory cytokine expression in the blood and potentiation of fibrosis in the tissues. In addition, CIITA-knockout mice maybe a good model to investigation of extracellular matrix expression and fibrosis in mice by mimicking the effect of CXCL4 on DC differentiation.

An important finding of our study is that CXCL4 alters the differentiation trajectory of monocyte derived dendritic cells. One may hypothesize that CXCL4-moDCs might actually be macrophages and not dendritic cells. We do not find any evidence supporting this hypothesis. Firstly, several studies have shown that LAMP1 and its family members LAMP2 and LAMP3 are abundantly expressed on moDCs (65, 66), taken their important function in antigen uptake, processing and presentation to T-cells. Figures 1G,H show that LAMP1 is abundantly expressed on day 6 in both conventional moDCs and CXCL4-moDCs. Secondly, several studies have reported that during differentiation of monocytes to moDCs the ability to uptake antigens and cell migration increases. When DCs mature, they lose their capacity for antigen uptake but strongly gain capacity of interacting with T-cells, along with the decline of the machinery involved in Ag uptake and cellular recognition and trafficking. This includes the down-regulation of CLEC10A, which plays crucial role in Ag uptake in immature DCs (67, 68). Taken our observations that CXCL4 strongly potentiates innate and adaptive immune responses, we postulate that CXCL4 drives cells toward maturation, and consequently decreases the expression of markers of immature DCs such as CLEC10A. As we expected, taken the observations by Higashi et al. (68) in moDCs and Heger et al. (67) in CD1c^+^ DCs, the expression of CLEC10A on CXCL4-moDCs before (Figure 1H) and after poly I:C stimulation (Figure S2D) is lower than in conventional moDCs before and after stimulation indicating the maturation promoted by CXCL4 during the differentiation. Thirdly, the classical macrophage markers such as CD163 and FCGR1A (CD64), are not expressed in differentiating CXCL4-moDCs and in conventional moDCs (data not shown). In addition, CD83, a dendritic cell marker, is highly expressed in both moDCs and even higher expressed in CXCL4-moDCs. In summary, although CXCL4 alters the differentiation trajectory of moDCs, CXCL4-moDCs do not resemble macrophages but are pro-inflammatory and pro-fibrotic moDCs.

In conclusion, our study reveals that differentiating monocytes undergo massive transcriptomic and epigenetic reprogramming upon CXCL4 exposure, and we propose that CXCL4 is a clinically relevant and important endogenous ligand bridging inflammation with fibrosis via trained immunity and provides a rationale for therapeutic targeting of CXCL4 in fibrotic diseases including SSc.

## Data Availability Statement

RNAseq count matrices and BAM files have been deposited in the National Center for Biotechnology Information's Gene Expression Omnibus under accession number GSE115488. Raw and processed DNA methylation data has been deposited in the National Center for Biotechnology Information's Gene Expression Omnibus under accession number GSE115201.

## Author Contributions

TR, MB, WT, and AP: conceptualization. SS-C, BG, MC, WT, CA, AL, and CB: methodology. WT, AD, and AP: formal analysis. TR: resources and project administration. SS-C, WT, AP, and TR: writing original draft. SS-C, WT, CA, AL, CB, AD, JL, WM, EH, RB, MB, AP, and TR: writing reviewing and editing. WT, SS-C, and AP: visualization. TR, MB, and AP: supervision. All authors contributed to the article and approved the submitted version.

## Conflict of Interest

The authors declare that the research was conducted in the absence of any commercial or financial relationships that could be construed as a potential conflict of interest.
