# Supplementary material for: CXCL4 Links Inflammation and Fibrosis by Reprogramming Monocyte-Derived Dendritic Cells in vitro
Source: Front Immunol. 2020 Sep 17;11:2149. doi: 10.3389/fimmu.2020.02149 (PMC7527415; doi:10.3389/fimmu.2020.02149)
Supplement: Supplementary file 1 [file Data_Sheet_1.pdf]

Supplementary Material

1 Supplementary Figures and Tables

1.1 Supplementary Figures

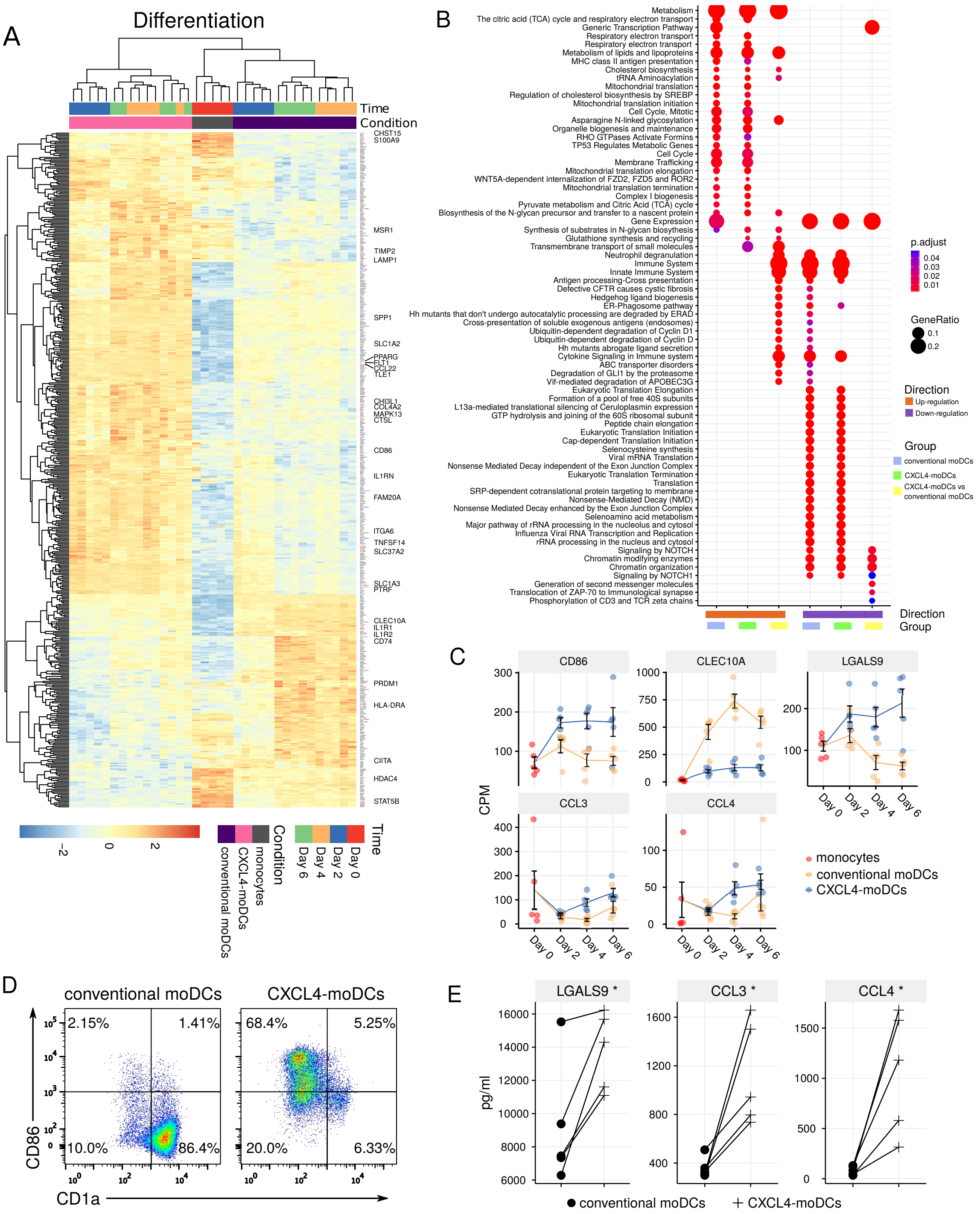

Figure S1. Differentially expressed genes during differentiation. (A) Heatmap showing normalized expression (VSD) of top 500 DEGs between CXCL4 moDCs and moDCs during their differentiation (day 0 to day 6). Heatmap color schemes are based on z-scores. Hierarchical clustering dendrograms were calculated using Euclidean distance. **(B)** Pathway enrichment analysis for the genes which were significantly up- and down-regulated during differentiation, as shown in **Figure 1B**. Up to 30 most significant pathways (FDR adjusted $p < 0.05$) were shown for each set of genes. The size of the circle depicts the gene ratio of DEGs in the pathway to the total number of DEGs in each set of genes. Circle color represents FDR adjusted p values. The full lists of pathways are available in **Table S5**. **(C)** Gene expression profiles for example genes from top 500 DEGs that were further validated at the protein level. Data are shown as mean of count per million (CPM) ± SEM. **(D)** Flow cytometry dot plot showing expression of CD86 and CD1a. Representative data from 5 HDs are shown. **(E)** Cytokine production of selected example proteins measured by Luminex using cell-free supernatants collected on day 6. Each symbol represents an individual donor; lines connect the same donor. Paired two-sided Student's *t*-test or two-sided Wilcoxon signed rank sum test (see statistical analyses in materials and methods).; * $P<0.05$.

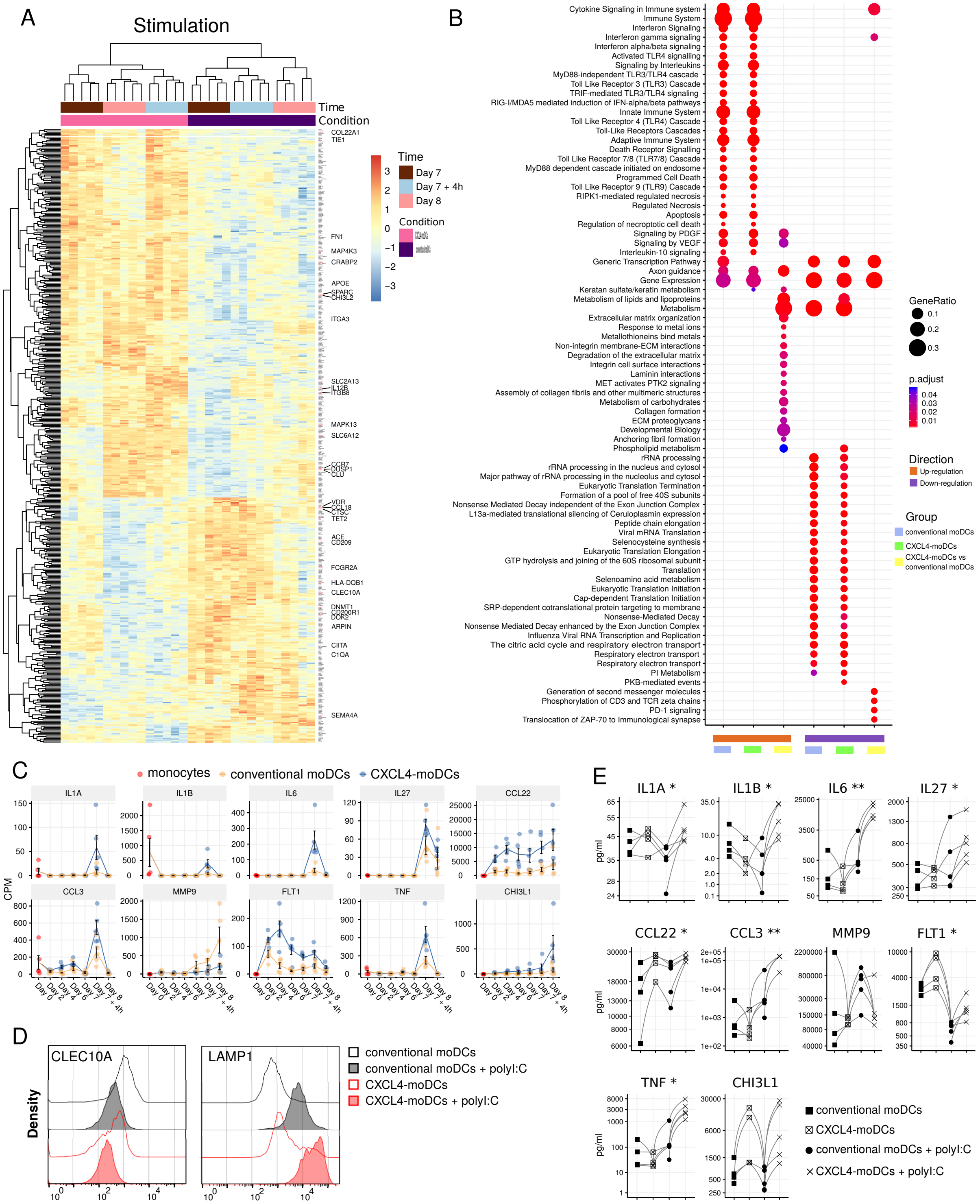

A
Stimulation
Time
Condition
Day 7
Day 7 + 4h
Day 8
COL22A1
TIE1
FN1
MAP4K3
CRABP2
APOE
SPARC
CHI3L2
ITGA3
SLC2A13
IL12B
ITGB8
MAPK13
SLC6A12
CCR7
DUSP1
CLU
VDR
CCL18
CTSC
TET2
ACE
CD209
FCGR2A
HLA-DQB1
CLEC10A
DNMT1
CD200R1
DOK2
ARPIN
CIITA
C1QA
SEMA4A
B
Cytokine Signaling in Immune system
Immune System
Interferon Signaling
Interferon gamma signaling
Interferon alpha/beta signaling
Activated TLR4 signalling
Signaling by Interleukins
MyD88-independent TLR3/TLR4 cascade
Toll Like Receptor 3 (TLR3) Cascade
TRIF-mediated TLR3/TLR4 signaling
RIG-I/MDA5 mediated induction of IFN-alpha/beta pathways
Innate Immune System
Toll Like Receptor 4 (TLR4) Cascade
Toll-Like Receptors Cascades
Adaptive Immune System
Death Receptor Signalling
Toll Like Receptor 7/8 (TLR7/8) Cascade
MyD88 dependent cascade initiated on endosome
Programmed Cell Death
Toll Like Receptor 9 (TLR9) Cascade
RIPK1-mediated regulated necrosis
Regulated Necrosis
Apoptosis
Regulation of necroptotic cell death
Signaling by PDGF
Signaling by VEGF
Interleukin-10 signaling
Generic Transcription Pathway
Axon guidance
Gene Expression
Keratan sulfate/keratin metabolism
Metabolism of lipids and lipoproteins
Metabolism
Extracellular matrix organization
Response to metal ions
Metallothioneins bind metals
Non-integrin membrane-ECM interactions
Degradation of the extracellular matrix
Integrin cell surface interactions
Laminin interactions
MET activates PTK2 signaling
Assembly of collagen fibrils and other multimeric structures
Metabolism of carbohydrates
Collagen formation
ECM proteoglycans
Developmental Biology
Anchoring fibril formation
Phospholipid metabolism
rRNA processing
rRNA processing in the nucleus and cytosol
Major pathway of rRNA processing in the nucleolus and cytosol
Eukaryotic Translation Termination
Formation of a pool of free 40S subunits
Nonsense Mediated Decay independent of the Exon Junction Complex
L13a-mediated translational silencing of Ceruloplasmin expression
Peptide chain elongation
Viral mRNA Translation
Selenocysteine synthesis
Eukaryotic Translation Elongation
GTP hydrolysis and joining of the 60S ribosomal subunit
Translation
Selenoamino acid metabolism
Eukaryotic Translation Initiation
Cap-dependent Translation Initiation
SRP-dependent cotranslational protein targeting to membrane
Nonsense-Mediated Decay
Nonsense Mediated Decay enhanced by the Exon Junction Complex
Influenza Viral RNA Transcription and Replication
The citric acid cycle and respiratory electron transport
Respiratory electron transport
Respiratory electron transport
PI Metabolism
PKB-mediated events
Generation of second messenger molecules
Phosphorylation of CD3 and TCR zeta chains
PD-1 signaling
Translocation of ZAP-70 to Immunological synapse
GeneRatio
0.1
0.2
0.3
p.adjust
0.04
0.03
0.02
0.01
Direction
Up-regulation
Down-regulation
Group
conventional moDCs
CXCL4-moDCs
CXCL4-moDCs vs conventional moDCs
C
monocytes
conventional moDCs
CXCL4-moDCs
IL1A
IL1B
IL6
IL27
CCL22
CCL3
MMP9
FLT1
TNF
CHI3L1
CPM
Day 0
Day 2
Day 4
Day 6
Day 7
Day 8
Day 7 + 4h
D
CLEC10A
LAMP1
Density
conventional moDCs
conventional moDCs + polyI:C
CXCL4-moDCs
CXCL4-moDCs + polyI:C
E
IL1A *
IL1B *
IL6 **
IL27 *
CCL22 *
CCL3 **
MMP9
FLT1 *
TNF *
CHI3L1
pg/ml
conventional moDCs
CXCL4-moDCs
conventional moDCs + polyI:C
CXCL4-moDCs + polyI:C

Figure S2. Differentially expressed genes upon polyI:C stimulation. (A) Heatmap same as **Figure S1** for top 500 DEGs between CXCL4 moDCs and moDCs upon polyI:C stimulation (day 7 to day 8). **(B)** Pathway enrichment analysis for the genes that were significantly up- and down-regulated upon polyI:C stimulation, shown in **Figure 1C**. Up to 30 most significant pathways (FDR adjusted $p < 0.05$) were shown for each set of genes. The size of the circle depicts the gene ratio of DEGs in the pathway to the total number of DEGs in each set of genes. Circle color represents FDR adjusted p values. The full lists of pathways are available in **Table S6**. **(C)** Gene expression profiles for example genes from top 500 DEGs that were further validated on protein level. Data are shown as mean of count per million (CPM) ± SEM. **(D)** Flow cytometry analysis showing the relative expression of CLEC10A and LAMP1 between CXCL4 moDCs and moDCs, unstimulated and stimulated with polyI:C for 24 hours (on day 8). Representative data from 5 HDs are shown. **(E)** Cytokine production in cell-free supernatants was measured by Luminex for cells with/without polyI:C stimulation for 24 hours on day 8. Each symbol represents an individual donor; lines connect the same donor. Paired two-sided Student's *t*-test or two-sided Wilcoxon signed rank sum test (see statistical analyses in materials and methods) was performed for "conventional moDCs + polyI:C" group and "CXCL4-moDCs + polyI:C" group; * $P<0.05$, ** $P < 0.01$.

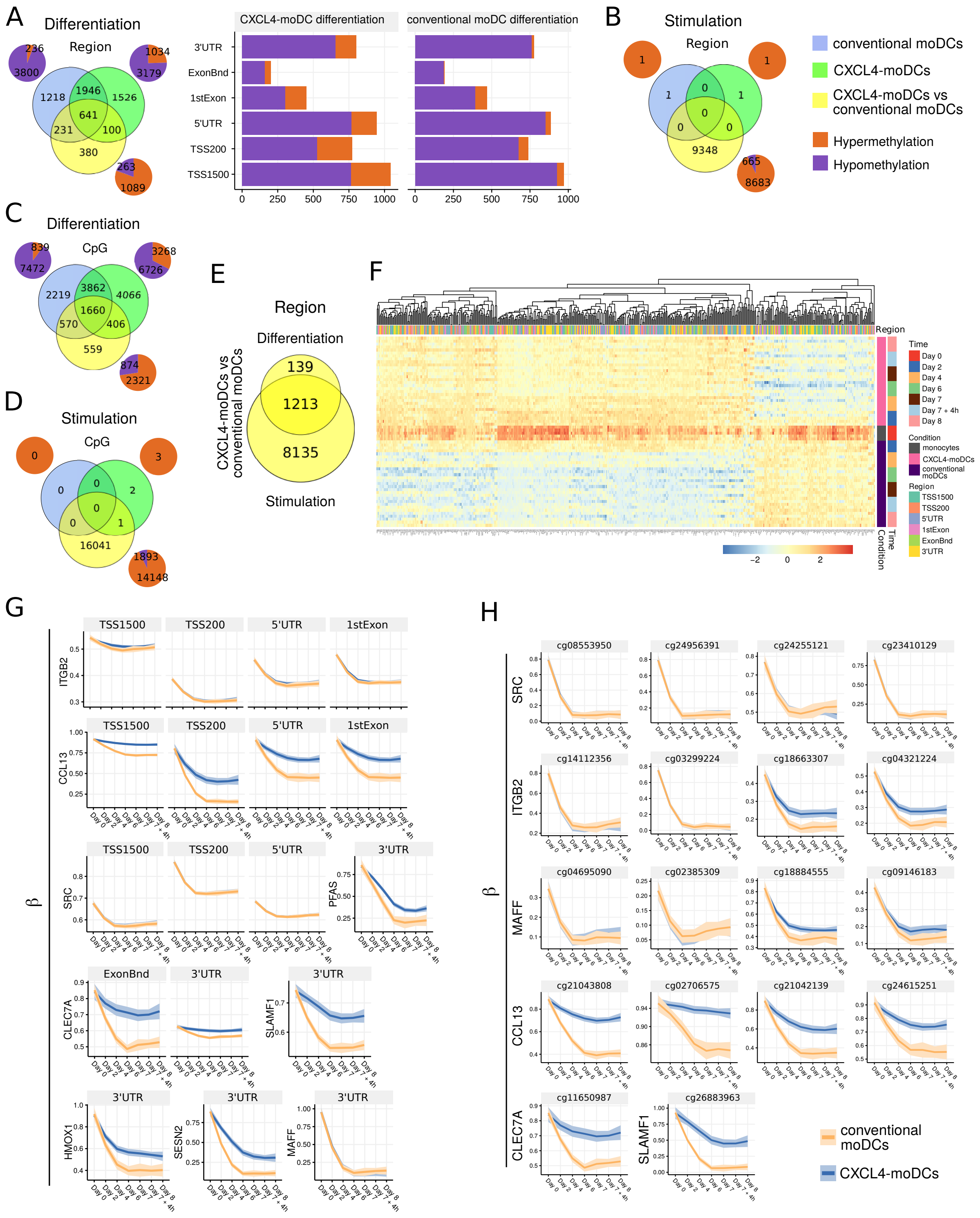
A
Differentiation
Region
236
3800
1034
3179
1218
1946
1526
641
231
100
380
263
1089
CXCL4-moDC differentiation
conventional moDC differentiation
3'UTR
ExonBnd
1stExon
5'UTR
TSS200
TSS1500
0 250 500 750 1000
B
Stimulation
Region
1
1
1
0
1
0
0
0
9348
665
8683
conventional moDCs
CXCL4-moDCs
CXCL4-moDCs vs conventional moDCs
Hypermethylation
Hypomethylation
C
Differentiation
CpG
839
7472
3268
6726
2219
3862
4066
1660
570
406
559
874
2321
D
Stimulation
CpG
0
3
0
0
2
0
0
1
16041
1893
14148
E
Region
Differentiation
CXCL4-moDCs vs conventional moDCs
139
1213
8135
Stimulation
F
Region
Time
Condition
Time
Day 0
Day 2
Day 4
Day 6
Day 7
Day 7 + 4h
Day 8
Condition
monocytes
CXCL4-moDCs
conventional moDCs
Region
TSS1500
TSS200
5'UTR
1stExon
ExonBnd
3'UTR
−2 0 2
G
β
ITGB2
CCL13
SRC
PFAS
CLEC7A
SLAMF1
HMOX1
SESN2
MAFF
TSS1500
TSS200
5'UTR
1stExon
3'UTR
ExonBnd
H
β
SRC
ITGB2
MAFF
CCL13
CLEC7A
SLAMF1
cg08553950
cg24956391
cg24255121
cg23410129
cg14112356
cg03299224
cg18663307
cg04321224
cg04695090
cg02385309
cg18884555
cg09146183
cg21043808
cg02706575
cg21042139
cg24615251
cg11650987
cg26883963
conventional moDCs
CXCL4-moDCs

Figure S3. Dynamics of DNA methylation on region and CpG levels. Venn diagram shows the overlaps of **(A)** differentially methylated regions (DMRs) and **(C)** CpG sites during differentiation of: monocytes into moDCs (blue); monocytes into CXCL4 moDCs (green), and DMRs between moDCs and CXCL4 moDCs (yellow). In the middle and right panels of **(A)**, bar charts show the distribution of DMRs across 6 regions during CXCL4-moDC differentiation (middle), and during conventional moDC differentiation (right), as a supplement of **Figure 2G**. (**B**) Venn diagram showing DMRs and CpG sites **(D)** in moDC (blue), CXCL4 moDCs (green) and DMR/CpG between moDCs and CXCL4 moDCs (yellow) after polyI:C stimulation. In **(A-D)** pie charts represent the number of hyper-methylated regions (orange) and hypo-methylated regions (magenta). **(E)** Venn diagram showing the overlap of DMRs during differentiation (yellow circle in panel **A**) and upon polyI:C stimulation (yellow circle in panel **B**). **(F)** Heatmap reporting top 500 regions in overlapping DMRs in panel **D**. **(G)** Temporal methylation patterns of selected regions and CpG sites **(H)** found to be differential in different comparisons. Lines represent mean of β values and shades represent 95% confidence interval.

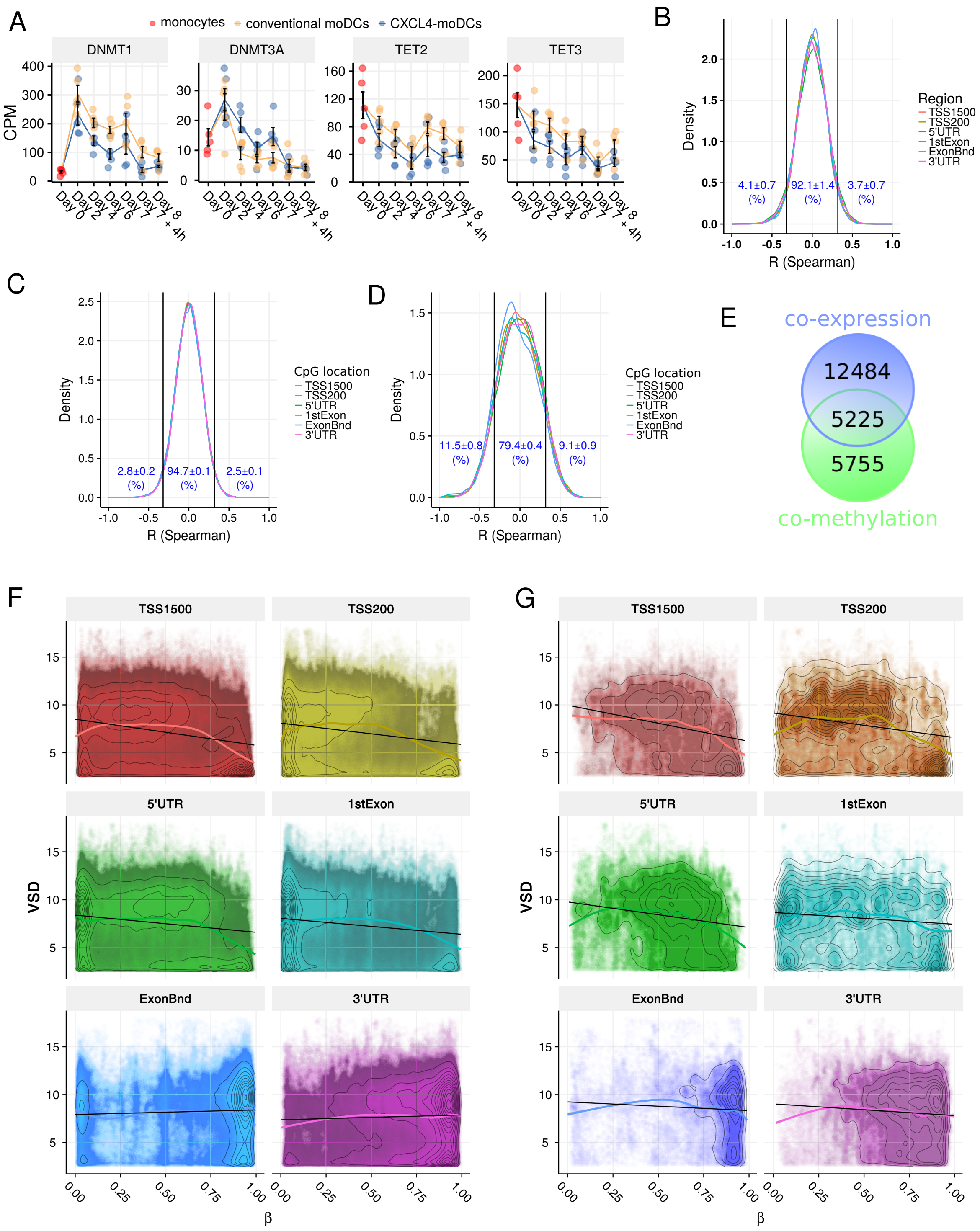
A
monocytes
conventional moDCs
CXCL4-moDCs
DNMT1
DNMT3A
TET2
TET3
CPM
Day 0
Day 2
Day 4
Day 6
Day 7
Day 8
+ 4h
B
Density
R (Spearman)
4.1±0.7 (%)
92.1±1.4 (%)
3.7±0.7 (%)
Region
TSS1500
TSS200
5'UTR
1stExon
ExonBnd
3'UTR
C
Density
R (Spearman)
2.8±0.2 (%)
94.7±0.1 (%)
2.5±0.1 (%)
CpG location
TSS1500
TSS200
5'UTR
1stExon
ExonBnd
3'UTR
D
Density
R (Spearman)
11.5±0.8 (%)
79.4±0.4 (%)
9.1±0.9 (%)
CpG location
E
co-expression
12484
5225
5755
co-methylation
F
G
TSS1500
TSS200
5'UTR
1stExon
ExonBnd
3'UTR
VSD
β

Figure S4. Comparison of transcriptome and DNA methylome. (A) Gene expression profile alterations of DNA methyltransferases and DNA demethylases. Data are shown as mean of count per million (CPM) ± SEM. **(B)** The distribution of Spearman correlation coefficients between the methylation levels of all regions (including the regions that are not differentially methylated) and the corresponding gene expression. **(C)** The distribution of Spearman correlation coefficients between the methylation levels of all CpG sites (including the CpGs that are not differentially methylated) and the corresponding gene expression. **(D)** The distribution of Spearman correlation coefficients between the methylation levels of differentially methylated CpGs and the corresponding gene expression. In (**B** to **D**) the cut-offs (two vertical lines at R = ±0.32) indicate significant correlation coefficients ($P < 0.01$). Overlap between differentially expressed genes (DEGs) and differentially methylated genes (DMGs) during differentiation and upon polyI:C stimulation. A gene is considered differentially methylated if there is at least one region within this gene that is differentially methylated. **(E)** Venn diagram shows the overlapping genes between DEGs and DMGs in all comparisons which were used for further analysis. **(F)** Contour plots show global comparison of β values (x-axis) and VSD values (y-axis) for all the genes. **(G)** Contour plots show global comparison of β values (x-axis) and VSD values (y-axis) for genes that are differentially expressed and methylated. The black straight lines were obtained by fitting a linear regression model and the smoothing curves were obtained by fitting a non-linear model (see Methods).

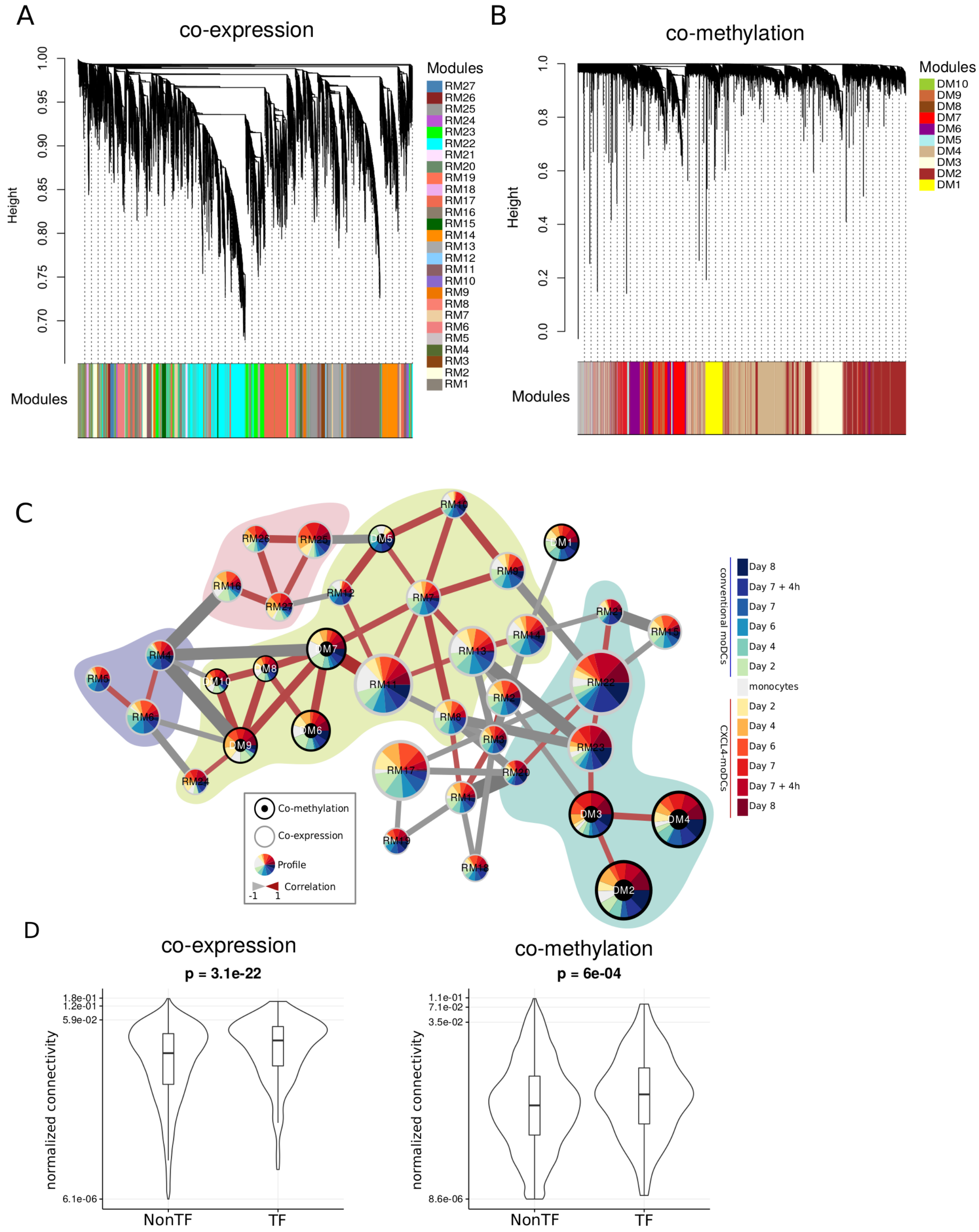
A
co-expression
Height
1.00
0.95
0.90
0.85
0.80
0.75
0.70
Modules
RM27
RM26
RM25
RM24
RM23
RM22
RM21
RM20
RM19
RM18
RM17
RM16
RM15
RM14
RM13
RM12
RM11
RM10
RM9
RM8
RM7
RM6
RM5
RM4
RM3
RM2
RM1
B
co-methylation
Height
1.0
0.8
0.6
0.4
0.2
0.0
Modules
DM10
DM9
DM8
DM7
DM6
DM5
DM4
DM3
DM2
DM1
C
Co-methylation
Co-expression
Profile
Correlation
-1
1
conventional moDCs
Day 8
Day 7 + 4h
Day 7
Day 6
Day 4
Day 2
monocytes
CXCL4-moDCs
Day 2
Day 4
Day 6
Day 7
Day 7 + 4h
Day 8
D
co-expression
p = 3.1e-22
normalized connectivity
1.8e-01
1.2e-01
5.9e-02
6.1e-06
NonTF
TF
co-methylation
p = 6e-04
normalized connectivity
1.1e-01
7.1e-02
3.5e-02
8.6e-06
NonTF
TF

Figure S5. Modules of co-expression and co-methylation networks. Hierarchical clustering dendrogram of genes generated using topological overlap matrix (TOM) obtained from **(A)** gene expression or **(B)** methylation data. The co-expression and co-methylation modules were obtained using WGCNA package and are shown with different colors independently. **(C)** Network of co-expression and co-methylation modules based on the correlation of module eigengenes. Each circle (node) represents either co-expression or co-methylation module. Co-methylation modules are denoted as a node with a black circle in the middle, while the other nodes denote co-expression modules. The size of each node depicts the number of genes within that module. Different time and conditions are represented by the colors shown in the legend. The colored pie chart within each node represents the eigengene profile of that module. The edges (lines between two nodes) represent spearman correlation coefficient (ρ) of eigengenes between two modules. The thickness of edge depicts the absolute value of ρ and edges with absolute value of $\rho<0.65$ are not shown. Colored shades in the background depict strongly positively correlated modules. **(D)** Violin/box plots comparing the connectivity, normalized by the total connectivity in the corresponding module, for transcription (co-)factors (TF) and other genes (NonTF) in the co-expression (left) and co-methylation (right) networks.

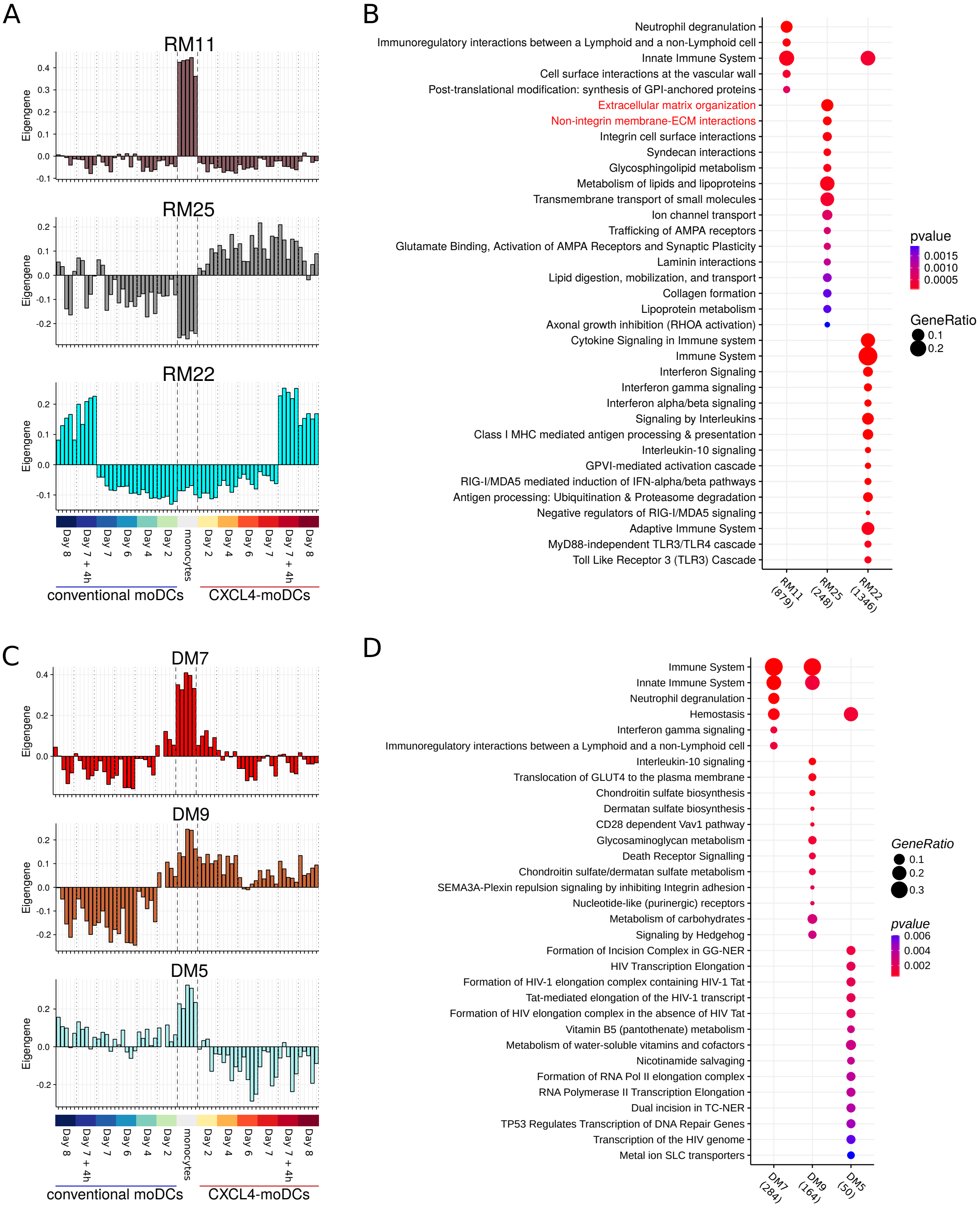
A
RM11
RM25
RM22
Eigengene
Day 8
Day 7 + 4h
Day 7
Day 6
Day 4
Day 2
monocytes
conventional moDCs
CXCL4-moDCs
B
Neutrophil degranulation
Immunoregulatory interactions between a Lymphoid and a non-Lymphoid cell
Innate Immune System
Cell surface interactions at the vascular wall
Post-translational modification: synthesis of GPI-anchored proteins
Extracellular matrix organization
Non-integrin membrane-ECM interactions
Integrin cell surface interactions
Syndecan interactions
Glycosphingolipid metabolism
Metabolism of lipids and lipoproteins
Transmembrane transport of small molecules
Ion channel transport
Trafficking of AMPA receptors
Glutamate Binding, Activation of AMPA Receptors and Synaptic Plasticity
Laminin interactions
Lipid digestion, mobilization, and transport
Collagen formation
Lipoprotein metabolism
Axonal growth inhibition (RHOA activation)
Cytokine Signaling in Immune system
Immune System
Interferon Signaling
Interferon gamma signaling
Interferon alpha/beta signaling
Signaling by Interleukins
Class I MHC mediated antigen processing & presentation
Interleukin-10 signaling
GPVI-mediated activation cascade
RIG-I/MDA5 mediated induction of IFN-alpha/beta pathways
Antigen processing: Ubiquitination & Proteasome degradation
Negative regulators of RIG-I/MDA5 signaling
Adaptive Immune System
MyD88-independent TLR3/TLR4 cascade
Toll Like Receptor 3 (TLR3) Cascade
RM11 (879)
RM25 (248)
RM22 (1346)
pvalue
0.0015
0.0010
0.0005
GeneRatio
0.1
0.2
C
DM7
DM9
DM5
Eigengene
Day 8
Day 7 + 4h
Day 7
Day 6
Day 4
Day 2
monocytes
conventional moDCs
CXCL4-moDCs
D
Immune System
Innate Immune System
Neutrophil degranulation
Hemostasis
Interferon gamma signaling
Immunoregulatory interactions between a Lymphoid and a non-Lymphoid cell
Interleukin-10 signaling
Translocation of GLUT4 to the plasma membrane
Chondroitin sulfate biosynthesis
Dermatan sulfate biosynthesis
CD28 dependent Vav1 pathway
Glycosaminoglycan metabolism
Death Receptor Signalling
Chondroitin sulfate/dermatan sulfate metabolism
SEMA3A-Plexin repulsion signaling by inhibiting Integrin adhesion
Nucleotide-like (purinergic) receptors
Metabolism of carbohydrates
Signaling by Hedgehog
Formation of Incision Complex in GG-NER
HIV Transcription Elongation
Formation of HIV-1 elongation complex containing HIV-1 Tat
Tat-mediated elongation of the HIV-1 transcript
Formation of HIV elongation complex in the absence of HIV Tat
Vitamin B5 (pantothenate) metabolism
Metabolism of water-soluble vitamins and cofactors
Nicotinamide salvaging
Formation of RNA Pol II elongation complex
RNA Polymerase II Transcription Elongation
Dual incision in TC-NER
TP53 Regulates Transcription of DNA Repair Genes
Transcription of the HIV genome
Metal ion SLC transporters
DM7 (284)
DM9 (164)
DM5 (50)
GeneRatio
0.1
0.2
0.3
pvalue
0.006
0.004
0.002

Figure S6. Characteristics of co-expression and co-methylation modules. (A) Bar charts show the eigengene of representative co-expression modules. **(B)** Pathway enrichment analyses for the modules shown in **(A)**. **(C)** Bar charts of the eigengene of representative co-mehtylation modules. **(D)** Enriched pathways analysis for the modules shown in **(C)**. In **(B)** and **(C)** the size of the circle depicts the gene ratio of DEGs in the pathway to the total number of DEGs in each set of genes; the colors of the circle represent FDR adjusted p values. In brackets, below the graph, we show the number of genes in each module. The full lists of pathways are available in **Table S8**.

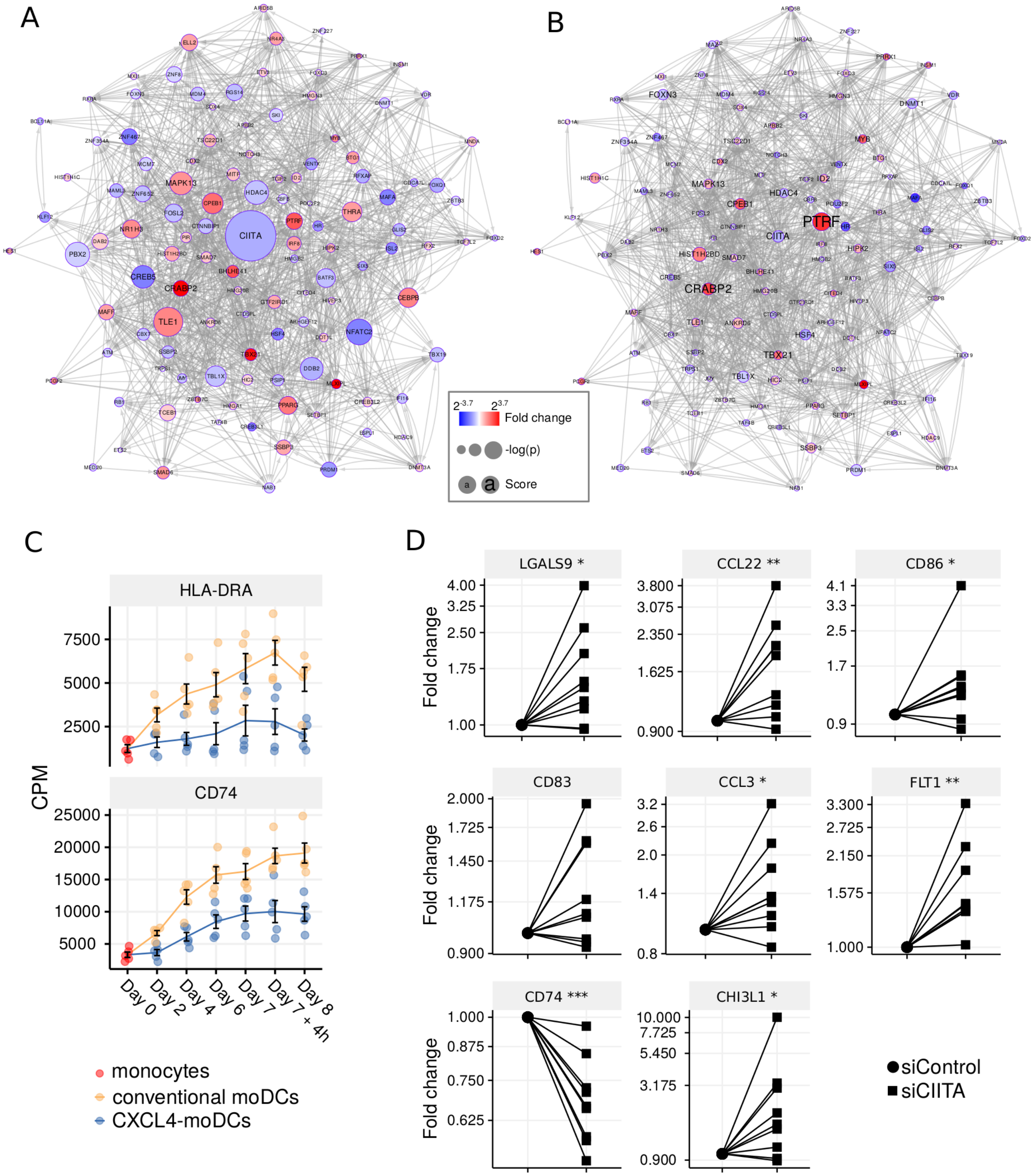
A
CIITA
MAPK13
HDAC4
CPEB1
PTRF
THRA
NR1H3
PBX2
CREB5
CRABP2
TLE1
CEBPB
NFATC2
DDB2
TBL1X
PPARG
B
FOXN3
PTRF
CIITA
CRABP2
TBX21
HSF4
2^{-3.7} 2^{3.7} Fold change
-log(p)
a a Score
C
HLA-DRA
CD74
CPM
7500
5000
2500
25000
20000
15000
10000
5000
Day 0
Day 2
Day 4
Day 6
Day 7
Day 7 + 4h
Day 8
monocytes
conventional moDCs
CXCL4-moDCs
D
LGALS9 *
CCL22 **
CD86 *
CD83
CCL3 *
FLT1 **
CD74 ***
CHI3L1 *
Fold change
4.00
3.25
2.50
1.75
1.00
3.800
3.075
2.350
1.625
0.900
4.1
3.3
2.5
1.7
0.9
2.000
1.725
1.450
1.175
0.900
3.2
2.6
2.0
1.4
0.8
3.300
2.725
2.150
1.575
1.000
1.000
0.875
0.750
0.625
10.000
7.725
5.450
3.175
0.900
siControl
siCIITA

Figure S7. Enriched transcription regulators obtained using random forest-based gene regulatory network. Enriched transcription regulator networks **(A)** during differentiation and **(B)** upon stimulation. Red indicates up- regulation and blue indicates down-regulation of TFs in CXCL4 moDCs compared to moDCs. Circle size shows ($-\log_{10}$(p)) obtained using differential expression analysis. Text size represents the overall score of TF enrichment obtained from RegEnrich (see Methods). Edges (lines) were obtained from top 5% edges in gene regulatory network inferred by random forest. To make the networks comparable same scales and parameters were used. **(C)** Gene expression profile of DEGs downstream to CIITA regulation. **(D)** After transfection of monocytes with Silencer negative control siRNA (siControl) and Silencer CIITA siRNA (siCIITA), cells were differentiated into moDCs for 6 days. On day 6, inflammatory and co-stimulatory genes were analyzed by qPCR. (Data are normalized by the mean expression of *RPL32* and *RPL13A*; fold change relative to siControl). Each symbol represents an individual moDC donor; lines connect the same donor. Paired two-sided Student's *t*-test or two-sided Wilcoxon signed rank sum test (see statistical analyses in materials and methods). * P <0.05, ** P < 0.01, *** P < 0.001.

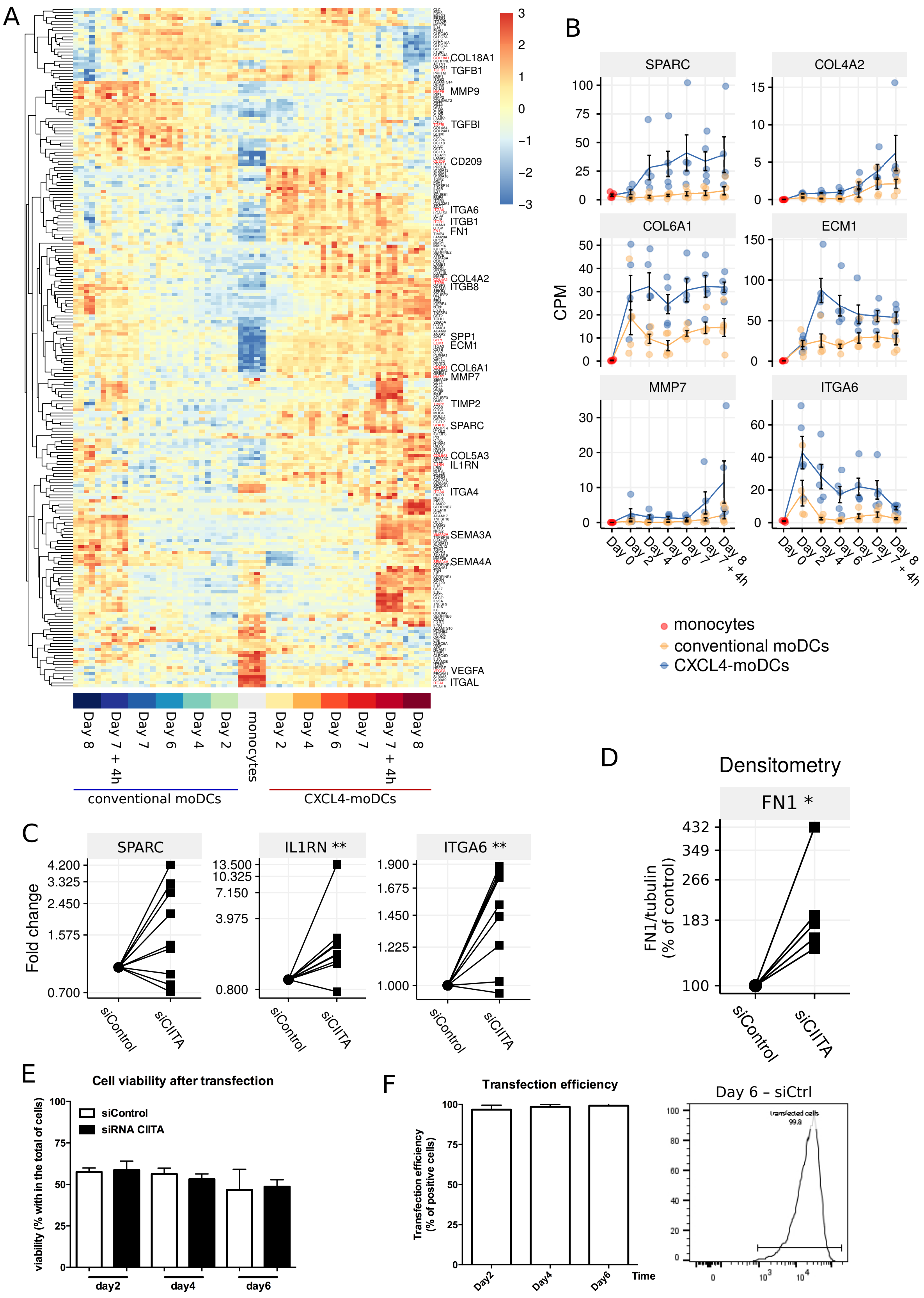
A
3
2
1
0
−1
−2
−3
COL18A1
TGFB1
MMP9
TGFBI
CD209
ITGA6
ITGB1
FN1
COL4A2
ITGB8
SPP1
ECM1
COL6A1
MMP7
TIMP2
SPARC
COL5A3
IL1RN
ITGA4
SEMA3A
SEMA4A
VEGFA
ITGAL
Day 8
Day 7 + 4h
Day 7
Day 6
Day 4
Day 2
monocytes
Day 2
Day 4
Day 6
Day 7
Day 7 + 4h
Day 8
conventional moDCs
CXCL4-moDCs
B
SPARC
COL4A2
COL6A1
ECM1
MMP7
ITGA6
CPM
Day 0
Day 2
Day 4
Day 6
Day 7
Day 7 + 4h
Day 8
monocytes
conventional moDCs
CXCL4-moDCs
C
SPARC
IL1RN **
ITGA6 **
Fold change
4.200
3.325
2.450
1.575
0.700
13.500
10.325
7.150
3.975
0.800
1.900
1.675
1.450
1.225
1.000
siControl
siCIITA
D
Densitometry
FN1 *
FN1/tubulin (% of control)
432
349
266
183
100
siControl
siCIITA
E
Cell viability after transfection
viability (% with in the total of cells)
siControl
siRNA CIITA
day2
day4
day6
F
Transfection efficiency
Transfection efficiency (% of positive cells)
Day2
Day4
Day6
Time
Day 6 – siCtrl
transfected cells
99.8

Figure S8. Identification of genes implicated in ECM remodeling. (A) Heatmap showing differentially expressed genes that play a role in ECM remodeling, identified from pathway enrichment analysis (**Figure S1B** and **Figure S2B**), and CXCL4 responsive co-expression modules (**Figure S5B**). Each column represents a sample and the colors on the bottom denote different time and conditions. The color schemes in the heatmap are shown as z-scores. **(B)** Expression profiles of example genes implicated in ECM remodeling. Data are shown as mean of count per million (CPM) ± SEM. **(C)** After transfection of monocytes with Silencer negative control siRNA (siControl) and Silencer CIITA siRNA (siCIITA), cells were differentiated into moDCs for 6 days. Genes involved in ECM remodeling/fibrosis analyzed by qPCR on day 6. Data are normalized by the mean expression of RPL32 and RPL13A; fold change relative to siControl. **(D)** Fibronectin (FN1) and tubulin expression measured by western blot on day 6. Signal intensity of 5 independent experiments was quantified by densitometry analysis. To determine the relative expression of FN1 between siControl and siCIITA, the ratio between the expression of FN1 and tubulin was first calculated. **(E-F)** Monocytes were transfected with either control non-targeting siRNA (siControl) or with specific siRNA targeting CIITA (siCIITA). Cells were differentiated into moDCs for 6 days. Viability (**E**) and transfection efficiency (**F**) was accessed by flow cytometry on days 2, 4 and 6. N=2. In panels **(C)** and **(D)** each symbol represents an individual moDC donor; lines connect the same donor. Paired two-sided Student's *t*-test or two-sided Wilcoxon signed rank sum test (see statistical analyses in materials and methods).. * P <0.05, ** P < 0.01, *** P < 0.001.

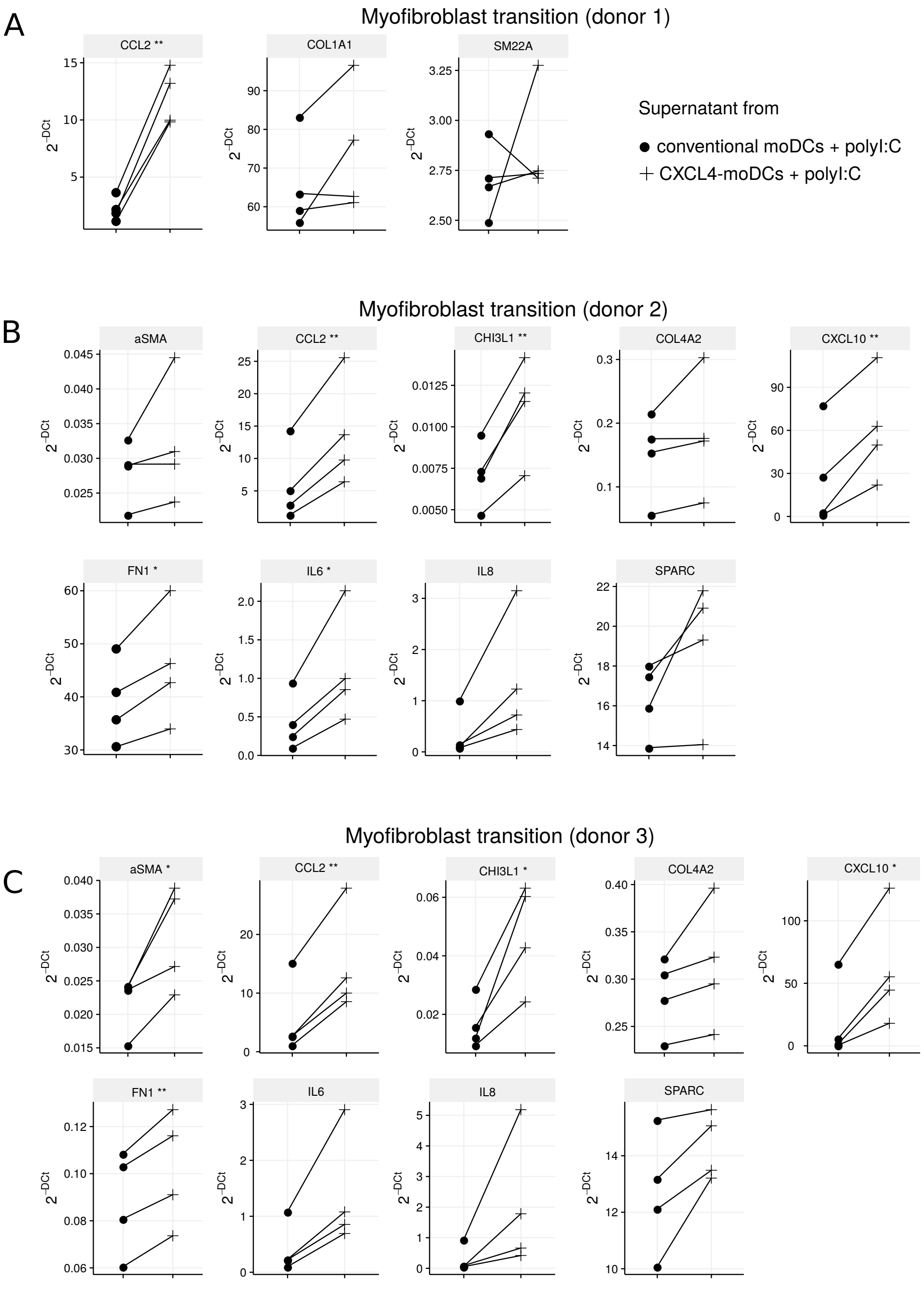
A
Myofibroblast transition (donor 1)
CCL2 **
COL1A1
SM22A
Supernatant from
conventional moDCs + polyI:C
CXCL4-moDCs + polyI:C
B
Myofibroblast transition (donor 2)
aSMA
CCL2 **
CHI3L1 **
COL4A2
CXCL10 **
FN1 *
IL6 *
IL8
SPARC
C
Myofibroblast transition (donor 3)
aSMA *
CCL2 **
CHI3L1 *
COL4A2
CXCL10 *
FN1 **
IL6
IL8
SPARC
2^-DCt

Figure S9. Gene expression analysis of healthy myofibroblasts after exposure to cell-free supernatants from CXCL4 moDCs and moDCs. CXCL4 moDCs and moDCs on day 7 were stimulated with polyI:C for 24 hours. Cell-free supernatants were added to healthy myofibroblasts for 24 hours. Inflammatory and fibrotic gene expression was analyzed by qPCR. Each symbol represents an individual moDC donor (n=4). Lines connect the same moDC donors. **(A)** shows the gene expression for the first fibroblast donor. The remaining measured genes are shown in **Figure 5H**. The gene expression for the **(B)** second and **(C)** third independent fibroblast donors. Paired two-sided Student's *t*-test or two-sided Wilcoxon signed rank sum test (see statistical analyses in materials and methods).. * $P < 0.05$, ** $P < 0.01$, *** $P < 0.001$.

1.2 Supplementary Tables

Table S1.

The number of differently expressed genes (DEGs) and differently methylated genes (DMGs) during differentiation and upon polyI:C stimulation.

Table S2.

Primer list for RT-qPCR.

Table S3.

DEGs and p-values by differential expression analysis.

Table S4.

DMRs and p-values by differential methylation analysis.

Table S5.

Full list of enriched pathways for the DEGs during differentiation.

Table S6.

Full list of enriched pathways for the DEGs upon stimulation.

Table S7.

Full list of enriched pathways for the DEGs overlapping during differentiation and upon stimulation.

Table S8.

Full list of enriched pathways for co-expression and co-methylation modules.

Table S9.

Transcription regulators used as TFs for the analysis.
